# Supplementary material for: Hypertension screening across different age groups in Indian adults: Evidence from nationally representative cross-sectional data
Source: PLoS One. 2025 Oct 29;20(10):e0332226. doi: 10.1371/journal.pone.0332226 (PMC12571314; doi:10.1371/journal.pone.0332226)
Supplement: Attachment — Submitted filename: Reviewers Comments PONE Paper.docx [file pone.0332226.s001.docx]

**Manuscript Title:**

*Hypertension Screening across Different Age Groups in Indian Adults: Evidence from Nationally Representative Cross-Sectional Data*

**Overall Evaluation:**

The manuscript presents a valuable study using **NFHS-5 data** to identify the optimal age for initiating hypertension screening in Indian adults. The findings are **highly relevant** for public health policymakers and contribute to the discourse on early detection of hypertension. The study employs robust statistical methods, including **multivariate logistic regression and ROC analysis**, to determine the recommended screening age of **35 years**.

While the manuscript is **methodologically sound**, revisions are necessary to improve clarity, statistical reporting, and alignment with journal guidelines. Below are **detailed comments and recommendations** to enhance the manuscript.

**Major Revisions Required**

**1. Introduction**

- The introduction should clearly **highlight the research gap** in India’s current hypertension screening policies and how this study aims to fill that gap.
- The authors should briefly discuss existing **global guidelines** on age-based hypertension screening and whether similar studies have been conducted in other **LMICs** (low- and middle-income countries).
- The rationale behind selecting **35 years** as the potential threshold should be better justified in light of previous research.

**2. Methods**

- **Ethical Considerations**: Although NFHS-5 data is publicly available, it is advisable to mention whether any **ethical approval** was sought for secondary data analysis or why it was not required.
- **Confounding Variables**: Were potential confounders (e.g., dietary habits, stress levels, family history of hypertension) excluded due to data limitations? If so, this should be acknowledged.
- **ROC Analysis**: The manuscript lacks detailed reporting on the **ROC curve analysis**. The authors should provide:
  - AUC (Area Under the Curve) values and their **confidence intervals**.
  - A **graphical representation** (ROC curve figure) to support the claim that **35 years** is the optimal threshold.
- **Data Handling & Missing Values**: Clarify whether missing data was handled via **imputation or exclusion** and whether it affected the results.

**3. Results**

- **Statistical Reporting:**
  - The results should explicitly mention **Odds Ratios (ORs) with Confidence Intervals (CIs)** for each predictor.
  - A **table summarizing the AUC values, sensitivity, and specificity** of different age thresholds should be included.
- **Figures and Tables:**
  - Consider adding a **visualization** of hypertension prevalence by age group.
  - If possible, include a **decision curve analysis** to illustrate the impact of different screening thresholds.

**4. Discussion**

- **Comparison with Existing Research:**
  - Strengthen the discussion by comparing findings with hypertension screening recommendations from organizations such as the **World Health Organization (WHO)** or **American Heart Association (AHA)**.
  - How do these findings align with or contradict previous Indian studies on hypertension screening?
- **Public Health and Policy Implications:**
  - The manuscript should provide **clear policy recommendations**. Should India’s national screening guidelines be revised to start at 35 years?
  - Are there specific **subpopulations** (e.g., rural vs. urban, male vs. female) that would benefit more from early screening?
- **Limitations:**
  - Acknowledge that **self-reported behaviors (e.g., alcohol, smoking, physical activity)** may be underreported in NFHS-5.
  - Discuss the **cross-sectional nature** of the study, limiting causal inferences.
  - Mention potential **regional disparities** in hypertension screening practices within India.

**5. Conclusion**

- The conclusion should **emphasize actionable recommendations** based on the study findings.
- The need for **longitudinal studies** to confirm the 35-year threshold should be mentioned.
- If available, suggest **intervention strategies** to improve screening uptake among younger adults.

**Minor Revisions & Language Edits**

- Ensure that all abbreviations (e.g., NFHS-5, AUC) are **defined upon first use**.
- Improve **flow and coherence** in the introduction and discussion sections.
- The manuscript would benefit from a **professional language edit** to enhance readability and conciseness.
