# Supplementary material for: Hypertension screening across different age groups in Indian adults: Evidence from nationally representative cross-sectional data
Source: PLoS One. 2025 Oct 29;20(10):e0332226. doi: 10.1371/journal.pone.0332226 (PMC12571314; doi:10.1371/journal.pone.0332226)
Supplement: Attachment — Submitted filename: PONE-D-25-01070_R1 (4).docx [file pone.0332226.s003.docx]

PLOS ONE

Hypertension Screening across Different Age Groups in Indian Adults: Evidence from

Nationally Representative Cross-Sectional Data

--Manuscript Draft--

| Manuscript Number: | PONE-D-25-01070R1 |
| --- | --- |
| Article Type: | Research Article |
| Full Title: | Hypertension Screening across Different Age Groups in Indian Adults: Evidence from Nationally Representative Cross-Sectional Data |
| Short Title: | Hypertension Screening and Risk Factors in Indian Adults |
| Corresponding Author: | Sonu Goel, Ph.D.  University of Limerick  Limerick, IRELAND |
| Keywords: | Hypertension Screening; Lifestyle factors; public health policy; Non-Communicable Diseases and Cardiovascular Risk |
| Abstract: | Background  Hypertension is a significant public health concern globally and in India, contributing substantially to the burden of cardiovascular diseases. Early detection through appropriate screening practices is critical to mitigating its long-term impact. However, there is limited evidence on age-specific screening practices for hypertension in the Indian population. This study aims to assess hypertension screening practices across different age groups and identify associated socio-demographic, behavioral, and physiological factors to inform effective public health strategies.  Methods  A cross-sectional study was conducted using data from the fifth National Family Health Survey (NFHS-5), which included 1,01,839 men and 7,24,115 women aged 15 years and above. Hypertension was defined based on systolic blood pressure (SBP) ≥140 mmHg and/or diastolic blood pressure (DBP) ≥90 mmHg. Multivariate logistic regression was used to analyze associations between hypertension and various sociodemographic and behavioral factors. Receiver Operating Characteristic (ROC) analysis determined the optimal age for initiating hypertension screening.  Results  The prevalence of hypertension increased linearly with age, with a significant rise observed in the 35-39 years age group. Factors significantly associated with hypertension included marital status, religion, education, wealth index, alcohol consumption, and waist-hip ratio. ROC analysis identified 35 years as the optimal age for initiating routine hypertension screening in both men and women. Screening in this age group showed balanced sensitivity and specificity for early detection of hypertension. Conclusions  This study highlights the need to revise hypertension screening policies in India, recommending initiation at 35 years to improve early detection and management. Addressing socio-demographic and lifestyle determinants through targeted interventions is critical to achieving national hypertension control goals. |
| Order of Authors: | Prakash Mathiyalagen |
|  | Anand Rajagopal |
|  | Kavita Vasudevan |
|  | Sridevi Gnanasekaran |
|  | Sharath BN |
|  | Jayanta Bora |
|  | Sonu Goel, Ph.D. |
| Response to Reviewers: | Manuscript Title:  Hypertension Screening across Different Age Groups in Indian Adults: Evidence from Nationally Representative Cross-Sectional Data |

|  | Major Revisions Required  1. Introduction  •The introduction should clearly highlight the research gap in India’s current hypertension screening policies and how this study aims to fill that gap.  •The authors should briefly discuss existing global guidelines on age-based hypertension screening and whether similar studies have been conducted in other LMICs (low- and middle-income countries).  •The rationale behind selecting 35 years as the potential threshold should be better justified in light of previous research.  Reviewer Comment 1:  Author Response:  We thank the reviewer for this insightful suggestion. In response, we have substantially revised the Introduction section to better highlight the policy and evidence gap in India’s current hypertension screening practices. Although the National Programme for  Prevention and Control of Cancer, Diabetes, Cardiovascular Diseases and Stroke  (NPCDCS) recommends annual hypertension screening for adults aged 30 years and above [1], this age threshold has not been empirically validated using stratified national data. Our study directly addresses this gap by analyzing data from NFHS-5, a nationally representative dataset, to determine an evidence-based optimal age for screening using Receiver Operating Characteristic (ROC) analysis.  To place our study in the global context, we have incorporated comparisons with international guidelines. The US Preventive Services Task Force (USPSTF) recommends annual screening for adults aged 40 years and above or for those at higher risk, and screening every 3 to 5 years for low-risk individuals aged 18–39 years  [2]. The World Health Organization (WHO) supports routine and opportunistic screening in primary care but does not define a specific age to begin [3]. The International Society of Hypertension (ISH) emphasizes personalized risk-based approaches rather than fixed age thresholds [4].  We also reviewed literature from other LMICs. A nationally representative study from  Nepal found significantly lower hypertension screening rates among individuals aged 15–29 years, indicating under-screening in younger adults and suggesting a missed opportunity for early diagnosis [5]. However, to our knowledge, no LMIC study has defined a data-driven, age-specific screening cutoff using ROC methods.  To justify the proposed threshold, we now clearly state in the revised manuscript that our analysis showed a marked and statistically significant increase in both systolic and diastolic blood pressure beginning at age 35. Furthermore, ROC analysis identified 36.5 years as the age with the most favorable balance of sensitivity and specificity based on the Youden index. These findings support the selection of 35 years as a practical and evidence-based starting point for routine hypertension screening in the Indian adult population. This recommendation can guide revisions to national policies and align hypertension control efforts with age-specific risk profiles.  References:  1.Ministry of Health and Family Welfare. Module for Multi-Purpose Workers –  Prevention, Screening and Control of Common NCDs. 2020. https://main.mohfw.gov.in 2.US Preventive Services Task Force. Screening for Hypertension in Adults: US Preventive Services Task Force Reaffirmation Recommendation Statement. JAMA.  2021;325(16):1650–1656.  3.World Health Organization. HEARTS Technical Package for Cardiovascular Risk Management in Primary Health Care: Risk-based CVD Management. Geneva: WHO; 2020.  4.Unger T, Borghi C, Charchar FJ, et al. 2020 International Society of Hypertension Global Hypertension Practice Guidelines. Hypertension. 2020;75(6):1334–1357.  5.Dhungana RR, Pedisic Z, Dhimal M, et al. Hypertension screening, awareness, treatment, and control: a study of their prevalence and associated factors in a nationally representative sample from Nepal. Global Health Action. 2022;15(1):2000092.  2. Methods  •Ethical Considerations: Although NFHS-5 data is publicly available, it is advisable to mention whether any ethical approval was sought for secondary data analysis or why it was not required.  •Confounding Variables: Were potential confounders (e.g., dietary habits, stress levels, family history of hypertension) excluded due to data limitations? If so, this should be acknowledged. |
| --- | --- |

|  | •ROC Analysis: The manuscript lacks detailed reporting on the ROC curve analysis.  The authors should provide:  oAUC (Area Under the Curve) values and their confidence intervals. oA graphical representation (ROC curve figure) to support the claim that 35 years is the optimal threshold.  •Data Handling & Missing Values: Clarify whether missing data was handled via imputation or exclusion and whether it affected the results.  Author Response:  We thank the reviewer for these constructive comments. In response, we have revised and elaborated upon the Methods and Results sections as follows: a) Ethical Considerations:  Although NFHS-5 is a publicly available and de-identified dataset, we have included a formal clarification in the Ethical Approval section to address this point. Specifically: “As this study involved secondary analysis of publicly available, anonymized data from the National Family Health Survey (NFHS-5), individual informed consent was not required. However, to ensure institutional compliance and uphold ethical standards, approval was obtained from the Institutional Ethics Committee of PGIMER, Chandigarh (Approval No. IEC-08/2022-2535, dated 17.08.2022).” b) Confounding Variables:  We acknowledge the absence of certain potentially important covariates in the NFHS-5 dataset. These have been explicitly acknowledged in the revised manuscript:  “Potential confounders such as dietary sodium intake, psychological stress levels, and family history of hypertension—well-known determinants of elevated blood pressure—were not collected in NFHS-5 and were therefore not included in the analysis. This limitation has been noted in the Discussion section.” c) ROC Analysis – Expanded Reporting:  We appreciate the recommendation to enhance the reporting of our ROC analysis. The manuscript now includes:  •AUC Values and Confidence Intervals:  “The ROC analysis yielded an AUC of 0.71 (95% CI: 0.70–0.72) for males and 0.68 (95% CI: 0.67–0.69) for females, indicating a fair ability of age to discriminate individuals with elevated systolic blood pressure (SBP).” •Youden Index and Threshold Justification:  “The Youden Index identified 36.5 years as the optimal age threshold, offering the best trade-off between sensitivity (57.9%) and specificity (49.7%) in men, with similar performance in women. These values support the recommendation of initiating hypertension screening at 35 years.” •ROC Curve Figures:  We have added Figures 5 and 6, which depict the ROC curves for males and females, respectively, and illustrate the diagnostic performance of age as a predictor of elevated BP.  d) Data Handling and Missing Values:  We have clarified our approach to missing data as follows:  “Data were analyzed using complete case analysis (listwise deletion). Participants with missing values in outcome or independent variables were excluded from relevant statistical models. No imputation was performed. The sample size varied slightly across analyses and has been reported in all relevant tables to ensure transparency.”  3. Results  •Statistical Reporting:  oThe results should explicitly mention Odds Ratios (ORs) with Confidence Intervals (CIs) for each predictor. oA table summarizing the AUC values, sensitivity, and specificity of different age thresholds should be included.  •Figures and Tables:  oConsider adding a visualization of hypertension prevalence by age group.  oIf possible, include a decision curve analysis to illustrate the impact of different screening thresholds.  Author Response:  We thank the reviewer for this valuable feedback. In response, we have made the following revisions and additions to strengthen the Results section:  Odds Ratios with 95% Confidence Intervals:  We now explicitly report adjusted Odds Ratios (ORs) along with their 95% Confidence  Intervals (CIs) for all significant predictors of elevated systolic and diastolic blood |
| --- | --- |

|  | pressure, as shown in the updated Tables 3 and 4. These findings are also summarized in the narrative text under the Results section to ensure clarity and interpretability.  Summary of ROC Metrics – Table 7:  We have added Table 7, which presents the key diagnostic performance metrics of age as a predictor of hypertension. This includes:  Table 7 presents the diagnostic performance of age as a predictor of hypertension using ROC analysis for both systolic and diastolic blood pressure in males and females. The analysis identifies 36.5 years as the optimal cut-off age for initiating hypertension screening in both sexes, based on the Youden Index. Among males, the area under the curve (AUC) was 0.71 for systolic BP and 0.69 for diastolic BP, indicating fair discriminatory ability. In females, the AUC values were slightly lower at 0.68 for systolic BP and 0.66 for diastolic BP. Sensitivity and specificity values at this cut-off were reasonably balanced, ranging from 56% to 61% sensitivity and 47% to 50% specificity. These findings support 35–36 years as a data-driven and practical threshold for routine hypertension screening among Indian adults.  Table 7: ROC-Based Diagnostic Performance of Age for Predicting Hypertension in Indian Adults (NFHS-5, 2019–21)  SexOutcomeCut-off Age (years)AUC (95% CI)Sensitivity (%)Specificity (%)Youden  Index  MaleSBP ≥140 mmHg36.50.71 (0.70–0.72)57.949.70.076  DBP ≥90 mmHg36.50.69 (0.68–0.70)56.550.20.067  FemaleSBP ≥140 mmHg36.50.68 (0.67–0.69)60.647.40.08 DBP ≥90 mmHg36.50.66 (0.65–0.67)57.847.60.054  Visual Trends in Blood Pressure by Age – Figures 1 to 4:  We have incorporated Figures 1–4 to visualize the age-wise trends in systolic and diastolic blood pressure separately for male and female participants. These figures clearly show a sharp increase in both SBP and DBP beginning from the age group of 35–39 years, reinforcing our empirical basis for selecting this threshold.  Decision Curve Analysis:  While we agree that decision curve analysis (DCA) can offer valuable insight into the clinical utility of screening thresholds, we were unable to perform DCA in the current analysis due to the cross-sectional nature and scope limitations of the NFHS-5 dataset, which does not capture longitudinal outcomes or decision-related consequences. However, we have acknowledged this limitation in the Discussion section and recommended DCA as a direction for future prospective cohort studies.  4. Discussion  •Comparison with Existing Research: oStrengthen the discussion by comparing findings with hypertension screening recommendations from organizations such as the World Health Organization (WHO) or American Heart Association (AHA). oHow do these findings align with or contradict previous Indian studies on hypertension screening?  •Public Health and Policy Implications: oThe manuscript should provide clear policy recommendations. Should India’s national screening guidelines be revised to start at 35 years? oAre there specific subpopulations (e.g., rural vs. urban, male vs. female) that would benefit more from early screening?  •Limitations: oAcknowledge that self-reported behaviors (e.g., alcohol, smoking, physical activity) may be underreported in NFHS-5. oDiscuss the cross-sectional nature of the study, limiting causal inferences. oMention potential regional disparities in hypertension screening practices within India.  Author Response:  We are grateful to the reviewer for this comprehensive and insightful feedback. In response, we have substantially revised the Discussion section to incorporate the following:  Comparison with Existing Guidelines and Research:  We have now elaborated on both global and national hypertension screening guidelines:  •The World Health Organization (WHO) promotes opportunistic and routine blood pressure screening in adults but does not prescribe a specific starting age, instead |
| --- | --- |

|  | emphasizing integration into primary care systems [1].  •The US Preventive Services Task Force (USPSTF) recommends annual hypertension screening for adults aged ≥40 years, or earlier for individuals with risk factors. For lowrisk adults aged 18–39, screening is advised every 3–5 years [2].  •In India, the NPCDCS guidelines advise annual screening starting at age 30 [3], but this threshold lacks direct support from empirical, age-stratified analyses using national-level data.  Our study fills this critical gap by identifying 36.5 years as the optimal age for initiating screening, based on ROC analysis of NFHS-5 data. This is the first Indian study to derive such a threshold from a nationally representative sample. Prior research, including the study by Prenissl et al. (2019), has highlighted poor awareness and control rates of hypertension in India but has not addressed the age at which screening should ideally begin [4]. Our findings thus provide a valuable addition to both Indian and global literature on evidence-based screening age thresholds.  Public Health and Policy Implications:  To translate these findings into actionable recommendations, we have revised the Discussion and Conclusion to include the following:  “Based on the ROC-derived thresholds and observed age-related trends in blood pressure, we recommend that India’s national guidelines revise the starting age for routine hypertension screening to 35 years for both males and females. This age captures the point at which blood pressure begins to rise substantially, and balances early detection with screening efficiency.”  Furthermore, we highlight the need for subpopulation-specific strategies:  •Urban males and adults with elevated waist-hip ratios exhibited significantly higher odds of hypertension and may benefit from earlier or more frequent screening intervals.  •Women with diabetes or obesity are another high-risk group for whom earlier intervention may be warranted.  •In rural and underserved regions, enhanced community outreach and mobile screening programs could help mitigate barriers to access and awareness.  Limitations:  The Limitations section has been expanded to include the following:  •“Self-reported variables in NFHS-5, including alcohol consumption, smoking, and physical activity, may be prone to social desirability bias and underreporting, potentially attenuating the observed associations.”  •“Due to the cross-sectional nature of NFHS-5, the study cannot establish causal relationships between risk factors and elevated blood pressure. Longitudinal studies are necessary to validate our proposed screening threshold.”  •“We acknowledge potential regional disparities in hypertension awareness, screening uptake, and access to care across Indian states and districts, which may affect the generalizability of our recommendations. Future subnational analyses are recommended.” References:  1.World Health Organization. HEARTS Technical Package: Risk-Based Cardiovascular Disease (CVD) Management. Geneva: WHO; 2020.  https://www.who.int/publications/i/item/9789240001367  2.US Preventive Services Task Force. Screening for Hypertension in Adults: US Preventive Services Task Force Reaffirmation Recommendation Statement. JAMA.  2021;325(16):1650–1656.  3.Ministry of Health and Family Welfare, India. Module for Multi-Purpose Workers – Prevention, Screening and Control of Common NCDs. 2020.  4.Prenissl J, Manne-Goehler J, Jaacks LM, et al. Hypertension screening, awareness, treatment, and control in India: A nationally representative cross-sectional study among individuals aged 15–49 years. PLoS Med. 2019;16(5):e1002801.  5. Conclusion  •The conclusion should emphasize actionable recommendations based on the study findings.  •The need for longitudinal studies to confirm the 35-year threshold should be mentioned.  •If available, suggest intervention strategies to improve screening uptake among younger adults.  Author Response: |
| --- | --- |

|  | We thank the reviewer for this important recommendation. In response, we have revised the Conclusion section to incorporate the following elements:  •Actionable Policy Recommendation:  We now emphasize the key takeaway that, based on nationally representative ROC analysis, hypertension screening should ideally begin at age 35 for both men and women in India. This represents a meaningful, evidence-based update to current guidelines that can enhance early detection and improve long-term cardiovascular outcomes.  •Need for Longitudinal Validation:  We have acknowledged that the cross-sectional nature of the NFHS-5 dataset limits causal inference and that longitudinal cohort studies are needed to further validate 35 years as the optimal screening threshold.  •Intervention Strategies:  We have added practical suggestions for improving screening uptake among younger adults, particularly: oCommunity-based awareness campaigns targeting the 30–45 age group. oIntegration of BP screening into workplace wellness programs. oMobile-based digital health reminders and use of ASHAs and frontline health workers to expand screening in underserved areas.  These additions aim to ensure the conclusion is policy-relevant, forward-looking, and aligned with public health implementation goals.  Minor Revisions & Language Edits  •Ensure that all abbreviations (e.g., NFHS-5, AUC) are defined upon first use.  •Improve flow and coherence in the introduction and discussion sections.  •The manuscript would benefit from a professional language edit to enhance readability and conciseness.  Thank you for the comments have modified it. |
| --- | --- |
| Additional Information: |  |
| Question | Response |
| Financial Disclosure  Enter a financial disclosure statement that describes the sources of funding for the work included in this submission. Review the [submission guidelines](http://journals.plos.org/plosone/s/submission-guidelines#loc-financial-disclosure-statement) for detailed requirements. View published research articles from [PLOS ONE](https://journals.plos.org/plosone/) for specific examples.  This statement is required for submission and will appear in the published article if the submission is accepted. Please make sure it is accurate. | The author(s) received no specific funding for this work. |

| Funded studies  Enter a statement with the following details:   - Initials of the authors who received each award - Grant numbers awarded to each author - The full name of each funder - URL of each funder website - Did the sponsors or funders play any role in the study design, data collection and analysis, decision to publish, or preparation of the manuscript? |  |
| --- | --- |
| Did you receive funding for this work? |  |
| Competing Interests  Use the instructions below to enter a competing interest statement for this submission. On behalf of all authors, disclose any [competing interests](http://journals.plos.org/plosone/s/competing-interests) that could be perceived to bias this work—acknowledging all financial support and any other relevant financial or nonfinancial competing interests.  This statement is required for submission and will appear in the published article if the submission is accepted. Please make sure it is accurate and that any funding sources listed in your Funding Information later in the submission form are also declared in your Financial Disclosure statement.  View published research articles from [PLOS ONE](http://journals.plos.org/plosone/) for specific examples. | none |

| NO authors have competing interests  Enter: The authors have declared that no competing interests exist.  Authors with competing interests  Enter competing interest details beginning with this statement:  I have read the journal's policy and the authors of this manuscript have the following competing interests: [insert competing interests here] |  |
| --- | --- |
| * typeset |  |
| Ethics Statement  Enter an ethics statement for this submission. This statement is required if the study involved:   - Human participants - Human specimens or tissue - Vertebrate animals or cephalopods - Vertebrate embryos or tissues - Field research   Write "N/A" if the submission does not require an ethics statement.  General guidance is provided below. Consult the [submission guidelines](http://journals.plos.org/plosone/s/submission-guidelines#loc-guidelines-for-specific-study-types) for detailed instructions. Make sure that all information entered here is included in the Methods section of the manuscript. | not applicable |

| Format for specific study types  Human Subject Research (involving human participants and/or tissue)   - Give the name of the institutional review board or ethics committee that approved the study - Include the approval number and/or a statement indicating approval of this research - Indicate the form of consent obtained (written/oral) or the reason that consent was not obtained (e.g. the data were analyzed anonymously)   Animal Research (involving vertebrate animals, embryos or tissues)   - Provide the name of the Institutional Animal Care and Use Committee (IACUC) or other relevant ethics board that reviewed the study protocol, and indicate whether they approved this research or granted a formal waiver of ethical approval - Include an approval number if one was obtained - If the study involved non-human primates, add additional details about animal welfare and steps taken to ameliorate suffering - If anesthesia, euthanasia, or any kind of animal sacrifice is part of the study, include briefly which substances and/or methods were applied   Field Research  Include the following details if this study involves the collection of plant, animal, or other materials from a natural setting:   - Field permit number - Name of the institution or relevant body that granted permission |  |
| --- | --- |
|  |  |
| Data Availability  Authors are required to make all data underlying the findings described fully available, without restriction, and from the time of publication. PLOS allows rare exceptions to address legal and ethical concerns. See the [PLOS Data Policy](http://journals.plos.org/plosone/s/data-availability) and [FAQ](http://journals.plos.org/plosone/s/data-availability#loc-faqs-for-data-policy) for detailed information. | Yes - all data are fully available without restriction |

| A Data Availability Statement describing where the data can be found is required at submission. Your answers to this question constitute the Data Availability Statement and will be published in the article, if accepted. |  |
| --- | --- |
| Important: Stating ‘data available on request from the author’ is not sufficient. If your data are only available upon request, select ‘No’ for the first question and explain your exceptional situation in the text box. |  |
| Do the authors confirm that all data underlying the findings described in their manuscript are fully available without restriction? |  |
| Describe where the data may be found in full sentences. If you are copying our sample text, replace any instances of XXX with the appropriate details.   - If the data are held or will be held in a public repository, include URLs, accession numbers or DOIs. If this information will only be available after acceptance, indicate this by ticking the box below. For example: All XXX files   are available from the XXX database (accession number(s) XXX, XXX.).   - If the data are all contained within the manuscript and/or Supporting Information files, enter the following: All relevant data are within the manuscript and its Supporting Information files. - If neither of these applies but you are able to provide details of access elsewhere, with or without limitations, please do so. For example:   Data cannot be shared publicly because of [XXX]. Data are available from the XXX Institutional Data Access / Ethics Committee (contact via XXX) for researchers who meet the criteria for access to confidential data.  The data underlying the results presented in the study are available from (include the name of the third party | This study is based on data from the Demographic and Health Surveys (DHS) Program (https://eur03.safelinks.protection.outlook.com/?url=https%3A%2F%2Fdhsprogram.co m%2F&data=05%7C02%7Csonu.goel%40ul.ie%7Cc6232b9bbc4a47e701c908dd9d47 accc%7C0084b9243ab4411692519939f695e54c%7C0%7C0%7C6388396533737667 20%7CUnknown%7CTWFpbGZsb3d8eyJFbXB0eU1hcGkiOnRydWUsIlYiOiIwLjAuMD AwMCIsIlAiOiJXaW4zMiIsIkFOIjoiTWFpbCIsIldUIjoyfQ%3D%3D%7C0%7C%7C%7C& sdata=oKOA3hBaB1e6FEdgI7d0Hu8ygQrSPw6e1UBsQmuJMzk%3D&reserved=0). At  the time of article publication, DHS data are no longer accessible due to a pause in operations resulting from an ongoing review of U.S. foreign assistance programs. The authors did not receive special access and obtained the data through standard procedures available to all registered researchers at that time. |

| and contact information or URL).  • This text is appropriate if the data are owned by a third party and authors do not have permission to share the data.  * typeset |  |
| --- | --- |
| Additional data availability information: | Tick here if the URLs/accession numbers/DOIs will be available only after acceptance of the manuscript for publication so that we can ensure their inclusion before publication.; Tick here if your circumstances are not covered by the questions above and you need the journal’s help to make your data available. |

Manuscript	[Click here to access/download;Manuscript;Manuscript](https://www2.cloud.editorialmanager.com/pone/download.aspx?id=40560272&guid=e7297c36-5ca2-430d-b9ff-16aabfe78b8e&scheme=1)

[Hypertension without track changes.docx](https://www2.cloud.editorialmanager.com/pone/download.aspx?id=40560272&guid=e7297c36-5ca2-430d-b9ff-16aabfe78b8e&scheme=1)

# Title: Hypertension Screening across Different Age Groups in Indian Adults: Evidence from Nationally Representative Cross-Sectional Data

Dr Prakash Mathiyalagen^1^, Dr Anand Rajagopal ^2^, Dr Kavita Vasudevan^3^, Dr Sridevi

Gnanasekaran^4,^Dr Sharath BN^5^, Dr Jayanta Bora ^6^, Dr Sonu Goel ^7&^

^1^Associate Professor, Dept of Community Medicine, IGMC&RI, Puducherry

^2^Assistant Professor, Dept of Community Medicine, IGMC&RI, Puducherry

^3^Professor & Head, Dept of Community Medicine, IGMC&RI, Puducherry

^4^Senior Resident, Dept of Community Medicine, IGMC&RI, Puducherry

^5^Faculty, ESIC Medical College, Bengaluru

^6^Founding & Executive Director, VART Consulting Pvt, Ltd, Delhi-NCR & Mumbai, India

^7^ Postgraduate Institute of Medical Education and Research (PGIMER), Chandigarh, India ^&^ Corresponding author

E-mail: sonugoel007@yahoo.co.in

# Background

Hypertension is a significant public health concern globally and in India, contributing substantially to the burden of cardiovascular diseases. Early detection through appropriate screening practices is critical to mitigating its long-term impact. However, there is limited evidence on age-specific screening practices for hypertension in the Indian population. This study aims to assess hypertension screening practices across different age groups and identify associated socio-demographic, behavioral, and physiological factors to inform effective public health strategies.

# Methods

A cross-sectional study was conducted using data from the fifth National Family Health Survey (NFHS-5), which included 1,01,839 men and 7,24,115 women aged 15 years and above. Hypertension was defined based on systolic blood pressure (SBP) ≥140 mmHg and/or diastolic blood pressure (DBP) ≥90 mmHg. Multivariate logistic regression was used to analyze associations between hypertension and various socio-demographic and behavioral factors. Receiver Operating Characteristic (ROC) analysis determined the optimal age for initiating hypertension screening.

# Results

The prevalence of hypertension increased linearly with age, with a significant rise observed in the 3539 years age group. Factors significantly associated with hypertension included marital status, religion, education, wealth index, alcohol consumption, and waist-hip ratio. ROC analysis identified 35 years as the optimal age for initiating routine hypertension screening in both men and women. Screening in this age group showed balanced sensitivity and specificity for early detection of hypertension. **Conclusions**

This study highlights the need to revise hypertension screening policies in India, recommending initiation at 35 years to improve early detection and management. Addressing socio-demographic and lifestyle determinants through targeted interventions is critical to achieving national hypertension control goals.

**Keywords:** Hypertension Screening, Lifestyle Factors, Public Health Policy, Non-Communicable Diseases and Cardiovascular Risk

**Introduction:**

Worldwide, the prevalence of hypertension among adults (30-79 years) in 2019 was 32% in women and 34% in men(1). Among those with hypertension, 41% of women and 51% of men did not have a previous diagnosis of hypertension(1). Less than half of the people with hypertension had taken treatment leading to the treatment rate of 47% in women and 38% in men(1). The hypertension control after taking treatment was achieved for 23% women and 18%men with hypertension(1).As per National Non-communicable Disease Monitoring Survey (NNMS) in India, the prevalence of hypertension among adults aged 18-69 years was 28.5%. Among them, 27% were aware of their hypertensive status, 14.5% were on treatment for hypertension and 12.6% had blood pressure under control(2).

Globally in 2019, high systolic blood pressure (SBP) was the leading risk factor for attributable deaths, accounting for 10.8 million deaths (19.2% of all deaths in 2019)(3). High SBP was attributed to 5.25 million (20.3%) and 5.60 million (18.2%) deaths among females and males respectively in 2019 (3). The leading risk factor in 2019 by percentage of Disability Adjusted Life Years (DALYs) was attributed to high SBP with 9.3 (8.2 to 10.5), 6.0 (4.9 to 7.1), 16.1 (14.2 to 18.0) and 19.5 (16.3 to 22.7) for all ages, 25-49 years, 50-74 years and 75 years and above respectively(3). In most parts of southeast Asia, 10% to 20% of DALYs were attributable to high SBP(3).

International Society of Hypertension (ISH) recommended that for those with normal blood pressure (BP) level (<130/85mmHg), BP would be re-measured after 1 year and 3 years in those with risk factors and without risk factors respectively(4). US Preventive Services Task Force (USPSTF) acknowledged the lack of evidence for optimal screening intervals and recommended the screening practice of once a year among those adults aged 40 and above and also in adults with risk factors and every 3-5 years among adults aged 18-39 years without any risk factors for hypertension(5). While several LMICs face a growing burden of hypertension, few have conducted large-scale, nationally representative studies to determine an age threshold for screening. A study in Nepal by Dhungana et al. (2022) noted that younger adults aged 15–29 years were significantly less likely to have been screened for hypertension, highlighting a missed opportunity for early detection in LMICs(6). However, this study did not provide a precise age cutoff for initiating screening. Our study is among the first in India and similar LMIC settings to use ROC analysis to empirically determine an optimal screening age.

In India, the recommendation under standard treatment guidelines for hypertension was to re-check BP in 2 years among persons with normal BP (<130/80mmHg) and in 1 year for persons with high normal BP(7). Under the National Programme for Prevention and Control of Cancer, DM, Cardiovascular Diseases and Stroke (NPCDCS), the recommended practice was to screen once a yearfor aged 30 years and above (8). Despite India's recommendation of initiating hypertension screening at 30 years under NPCDCS, there is no age-stratified evidence to support this threshold from nationally representative datasets. There is a need for sensitizing the health care providers to screen for high BP at optimum intervals without the need for unnecessary screening and overburdening them(9).

Among hypertensive patients, only 76% had ever received a BP screening according to NFHS-4(10) and 64% had not checked their BP in the last one year in Puducherry, India(11). In Nepal, the prevalence of hypertension screening was 65.9% among the hypertensive individuals(12). Hypertension screening was significantly lower among those in the age group of 15 to 29 years

(52.2%), those who were never married (54.4%) and those with no formal schooling (59.6%)(12). In Germany, 37.5% of men and 21.9% of women did not have BP measurement within the last one year among non hypertensive individuals(13). In Canada, 2.6% of the individuals did not have BP measurement ever and 9.0% did not have their BP assessed in the last 2 years(14). In America, 87.4% of individuals of aged 18 to 39 years have never measured their BP at home(13). Even though there is a policy for regular BP measurement with every country(13), there is a gap in the implementation of these policies.

Evidence suggests that SBP increases along with age in a linear relationship whereas DBP increases before 45 years of age and then decreases afterwards in a curvilinear fashion(15). However the relationship between and age varies from one country to another depending on the contributing factors(15). Hence there is a need to identify the relationship between age and BP and to find the appropriate age at which the screening is required for early diagnosis.

The Government of India has adopted a national multi-sectoral action plan for the prevention and control of non-communicable diseases and set a target of 25% relative reduction in the prevalence of raised blood pressure by 2025(16). Since hypertension is an asymptomatic condition in most of the individuals, it can increase the overall risk of cardio-vascular diseases (CVD) and lead to life threatening complications like heart attack, stroke and renal failure(7). Early diagnosis of high BP is achieved by appropriately screening the people at adequate intervals. Screening for high BP at an appropriate interval and at an appropriate age will not only help the individuals to lower the CVD risk factors but also in attaining the Government’starget towards reducing the high BP prevalence in the entire community.

There is a paucity of evidence to suggest the appropriate screening practice for a particular age group from a nation-wide representative sample, although there are some recommendations existing depending on the blood pressure values and for all age groups in general. In this regard, the present study has been planned with the objective of determining the hypertension screening practice across different age groups among the Indian population from a nationwide representative sample (NFHS5).The objectives of the current study were to assess the hypertension screening practice across different age groups in Indian adults and to identify the factors associated with hypertension among Indian adults

# Materials and Methods

*Study design:*

This cross sectional study utilizes secondary data from the fifth National Family Health Survey (NFHS-5), which is the Indian version of the Demographic and Health Survey conducted regularly in more than 90 countries worldwide. NFHS is a collaborative project supported by the International Institute of Population Sciences (IIPS) in Mumbai, India; International Classification of Functioning, Disability and Health (ICF) in Calverton, Maryland, USA, and East West Center in Honolulu, Hawaii,

USA. The Ministry of Health and Family Welfare (MoHFW) of the Government of India has appointed IIPS as the coordinating and technical guidance agency of NFHS. The survey is funded by the United States Agency for International Development (USAID) with additional support from the

United Nations Children’s Fund (UNICEF). NFHS uses four different types of questionnaires such as household, man’s, woman’s and biomarker in 19 languages using the Computer Assisted Personal Interviewing (CAPI). The fieldwork for NFHS-5 in India was done in two phases. Phase one took place between June 17, 2019, and January 30, 2020, which covered 17 states and 5 Union Territories. Phase two was conducted between January 2, 2020 and April 30, 2021, which covered 11 states and 3 Union Territories. A total of 17 Field Agencies gathered data from 6,36,699 households, 1,01,839 men and 7,24,115 women.

## Data source

The current data analysis uses the men’s questionnaire and women’s questionnaire from NFHS- 5, mainly focusing on male and female datasets. It focuses on the various independent factors associated with hypertension (dependent variable) among Indian adults.Analyzing the male and female factors separately can provide a clear understanding on the social and cultural factors associated with hypertension. Potential confounding variables such as dietary sodium intake, psychological stress, and family history of hypertension were not included due to limitations in the NFHS-5 data collection instruments. It examines the ideal age points at which the Indian adult population has to be screened for hypertension. Usually, the age cut off for screening hypertension has been taken as 30 years and the evidence provided through a nationwide survey would be more convincing regarding the appropriate age cut off. The total sample size utilized for this secondary data analysis was 1,01,839 men and 7,24,115 women aged 15 years and above. Since there are missing values, each variable in this analysis has a different sample size, as mentioned in the results section.

## Ethical approval

The research was conducted using a pre-existing, anonymised data from a public source, which did not require direct involvement of the patients or the general public. Since the dataset did not contain any personally identifiable information about the study participants, a formal ethics committee approval was not mandatory. However, the ethics committee approval was obtained from the Institutional Ethics Committee of the Post Graduate Institute of Medical Education and Research (PGIMER) in Chandigarh (approval number IEC-08/2022-2535, dated 17.08.2022).

## Definitions

As per the guidelines of World Health Organisation (WHO) and American Heart Association (AHA), the study describes elevated blood pressure as having a systolic blood pressure of 140 mmHg or higher and/or a diastolic blood pressure of 90 mmHg or more(17). The raised risk of metabolic complications was considered when the waist-hip ratio (WHR) was 0.90 or higher for men and 0.85 or higher for women(18). Regions were divided into 6 categories namely north (Chandigarh, Delhi, Haryana, Himachal Pradesh, Jammu & Kashmir, Punjab, Rajasthan, Uttarakhand), central (Chattisgarh, Madhya Pradesh, Uttar Pradesh), east (Bihar, Jharkhand, Odisha and West Bengal), northeast (Arunachal Pradesh, Assam, Manipur, Meghalaya, Mizoram, Nagaland, Sikkim and Tripura), west (Dadra & Nagar Haveli, Daman & Diu, Goa, Gujarat, Maharashtra) and south (Andaman & Nicobar Islands, Andhra Pradesh, Karnataka, Kerala, Lakshadweep, Puducherry, Tamil Nadu and Telangana).

## Statistical analysis

Hypertension was identified as the outcome variable in the study. The average of three blood pressure readings that were collected during the NFHS was considered for the study. The quantitative blood pressure values were then converted into a dichotomous variable as presence or absence of hypertension. Sampling weights were incorporated into the data to address the differential probabilities of selection and participation.

Statistical analysis was performed using IBM SPSS version 20. The data were presented in the form of mean with 95% confidence interval (CI), frequency and percentage. The associations were evaluated using multivariable logistic regression analysis to calculate the adjusted odds ratio and 95% CI of raised blood pressure with age category (8 categories), marital status, religion, educational status, wealth index (5 categories), occupation, residence, region (6 categories), caste, waist-hip ratio, alcohol use, tobacco use, fruit intake, fried food intake and co-morbidities. The fitness of the dataset was assessed using the Hosmer and Lemeshow test and the Goodness of Fit test. Receiver Operating Characteristics (ROC) Curve was used to evaluate and compare the performance of classification models (age) where the outcome variable is binary (raised blood pressure: present/absent). Sensitivity and specificity was used to describe the diagnostic ability of the screening test. The Youden index was used as a summary measure of the ROC curve to select the optimal cut off point for the age within the age group.A p value of less than 0.05 was considered as significant. Missing data were handled by listwise deletion. Participants with missing values for outcome or explanatory variables were excluded from respective analyses. No imputation was performed. Sample sizes for each analysis are reported in the tables to reflect variations due to missing data.

**Results:**

A total of 1,01,839 men and 7,24,115 women participants were included in the NFHS-5 study. Since the missing values were excluded from the study, the sample size varied across different variables. Table 1 and Table 3 highlight the key findings from adult male population. The systolic and diastolic blood pressure increases with increasing age as shown in Figure 1 and Figure 2, which is also evident from the statistically significant trends in the increasing odds ratio. Though SBP does not have any association with marital status, the DBP is showing a significant association with marital status. Married men are having an increased risk of 1.12 times for developing raised DBP compared to never married men. Muslim men are 10% and 14% lesser risk of developing raised SBP and DBP respectively, compared Hindu men. Christian men are 11% lesser risk of developing raised DBP whereas Others category (Sikh, Buddhist, Jain and Jewish) men are having 1.29 times higher risk of developing raised SBP compared to Hindu men. With an increase in educational status, the odds of developing raised SBP and DBP also increases. When compared to no education men, the primary, secondary and higher educated men show 1.14,1.20 and 1.01 times of having higher SBP. The primary, secondary and higher educated men show 1.09, 1.24 and 1.12 times of having higher DBP in comparison with no education men. There is a linear trend in wealth index for having raised SBP and DBP. Richer and richest quintile shows 1.12 and 1.14 times of higher risk of developing raised SBP compared to poorest quintile. Poorer, middle, richer and richest quintile shows 1.17, 1.32, 1.32 and 1.35 times respectively of developing raised DBP compared to poorest quintile. Though the employed men has 5% and 3% lesser risk of developing raised SBP and DBP, the association was not found to be statistically significant. The rural men have 8% lower risk of developing raised SBP and DBP compared to urban men. Men belonging to east zone have 34% and 33% lesser risk of developing raised SBP and DBP respectively, compared to men from north zone. West zone has 25% lesser risk of raised SBP whereas south zone has 1.27 times higher risk of raised DBP. Men belonging to ST caste have 1.15 times higher risk developing raised SBP whereas OBC and Others has 11% and 9% lesser risk of developing raised DBP respectively.

**Figure 1: Male systolic blood pressure across different age group in the study population**

# Figure 2: Male diastolic blood pressure across different age group in the study population

The men having WHR more than 0.90 have 1.46 and 1.62 times higher risk of developing raised SBP and DBP respectively. Men having a history of alcohol consumption have 1.28 and 1.50 times higher risk of developing raised SBP and DBP respectively. Men having a history of tobacco use 1.20 and 1.05 times higher risk of developing raised SBP and DBP respectively, though the association between raised DBP and tobacco use was not significant. Regarding fruit intake, daily, weekly and occasionally consuming men have 20%. 9% and 15% reduced risk of developing raised DBP respectively, compared to never category men. Regarding fried food intake, daily consuming men have 1.18 times increased risk of developing raised SBP, compared to never category men. Daily, weekly and occasional consumers of fried food have 10%, 9% and 7% reduced risk of developing raised DBP respectively, compared to never category men. There is no association observed between men with co-morbidities such as diabetes and cardio-vascular diseases and raised SBP and DBP.

# Table 1: Distribution of systolic and diastolic blood pressure among males according to sociodemographic, behavioral and physiological characteristics of the study population, NFHS-5, 2019-21, India (N=1,01,839)

| **Characteristics** | **N** | **(%)** | **Systolic blood pressure** | | **Diastolic blood pressure** | |
| --- | --- | --- | --- | --- | --- | --- |
|  |  |  | **Weighted mean** | **95%CI** | **Weighted mean** | **95%CI** |
| **Socio-demography** |  |  |  |  |  |  |
| **Age category (in years)** |  |  |  |  |  |  |
| 15-19 | 14337 | 14.1 | 115.09 | 114.91-115.28 | 72.86 | 72.72-73.01 |
| 20-24 | 12473 | 12.2 | 119.14 | 118.95-119.34 | 76.53 | 76.37-76.68 |
| 25-29 | 12410 | 12.2 | 120.68 | 120.49-120.88 | 79.04 | 78.89-79.19 |
| 30-34 | 11592 | 11.4 | 122.63 | 122.41-122.84 | 81.00 | 80.84-81.17 |
| 35-39 | 11641 | 11.4 | 123.55 | 123.31-123.78 | 82.25 | 82.08-82.43 |
| 40-44 | 9667 | 9.5 | 124.96 | 124.66-125.25 | 83.52 | 83.31-83.72 |
| 45-49 | 10058 | 9.9 | 126.79 | 126.47-127.10 | 84.10 | 83.89-84.30 |
| 50-54 | 7947 | 7.8 | 128.67 | 128.30-129.03 | 84.57 | 84.34-84.80 |
| **Marital status** |  |  |  |  |  |  |
| Never married | 31756 | 31.2 | 118.00 | 117.87-118.13 | 75.75 | 75.65-75.85 |
| Currently married | 57031 | 56.0 | 124.22 | 124.11-124.34 | 82.16 | 82.08-82.24 |
| Widowed /Separated  /Divorced | 1337 | 1.3 | 125.24 | 124.42-126.06 | 82.65 | 82.11-83.19 |
| **Religion** |  |  |  |  |  |  |
| Hindu | 72249 | 70.9 | 122.10 | 122.00-122.20 | 80.03 | 79.95-80.10 |
| Muslim | 13122 | 12.9 | 121.16 | 120.94-121.39 | 78.83 | 78.67-79.00 |
| Christian | 2484 | 2.4 | 123.22 | 122.68-123.76 | 80.87 | 80.48-81.26 |

| Others* | 2269 | 2.2 | 124.09 | 123.48-124.69 | 81.27 | 80.83-81.70 |
| --- | --- | --- | --- | --- | --- | --- |
| **Educational status** |  |  |  |  |  |  |
| No education | 10893 | 10.7 | 122.95 | 122.68-123.22 | 80.93 | 80.73-81.12 |
| Primary | 10990 | 10.8 | 122.83 | 122.57-123.10 | 80.65 | 80.46-80.84 |
| Secondary | 51460 | 50.5 | 121.58 | 121.46-121.70 | 79.45 | 79.36-79.54 |
| Higher | 16781 | 16.5 | 122.38 | 122.18-122.57 | 80.17 | 80.03-80.32 |
| **Wealth Index of the household** | |  |  |  |  |  |
| Poorest (I quintile) | 15204 | 14.9 | 120.28 | 120.07-120.49 | 78.18 | 78.02-78.33 |
| Poorer (II quintile) | 17872 | 17.5 | 121.21 | 121.00-121.41 | 79.16 | 79.01-79.31 |
| Middle (III quintile) | 19497 | 19.1 | 122.03 | 121.83-122.22 | 80.08 | 79.94-80.23 |
| Richer (IV quintile) | 20190 | 19.8 | 122.76 | 122.57-122.95 | 80.67 | 80.53-80.81 |
| Richest (V quintile) | 17362 | 17.0 | 123.64 | 123.43-123.84 | 81.12 | 80.96-81.27 |
| **Current occupation** |  |  |  |  |  |  |
| Unemployed | 21208 | 20.8 | 118.27 | 118.10-118.45 | 76.18 | 76.05-76.31 |
| Employed | 68917 | 67.7 | 123.20 | 123.10-123.31 | 81.06 | 80.98-81.13 |
| **Residence** |  |  |  |  |  |  |
| Urban | 30569 | 30.0 | 122.84 | 122.68-122.99 | 80.66 | 80.55-80.77 |
| Rural | 59555 | 58.5 | 121.64 | 121.53-121.75 | 79.52 | 79.44-79.61 |
| **Region** |  |  |  |  |  |  |
| North | 7722 | 7.6 | 124.57 | 124.28-124.87 | 80.90 | 80.68-81.11 |
| Central | 10302 | 10.1 | 123.21 | 122.95-123.46 | 80.31 | 80.12-80.51 |
| East | 23013 | 22.6 | 120.36 | 120.19-120.53 | 78.28 | 78.15-78.40 |
| Northeast | 5000 | 4.9 | 122.87 | 122.49-123.25 | 80.16 | 79.88-80.43 |
| West | 21571 | 21.2 | 121.28 | 121.10-121.46 | 79.70 | 79.56-79.83 |
| South | 22516 | 22.1 | 122.93 | 122.74-123.12 | 81.20 | 81.06-81.35 |
| **Caste** |  |  |  |  |  |  |
| SC | 18473 | 18.1 | 122.04 | 121.83-122.23 | 80.05 | 79.90-80.20 |
| ST | 8366 | 8.2 | 122.37 | 122.07-122.66 | 80.01 | 79.79-80.23 |
| OBC | 38188 | 37.5 | 121.78 | 121.64-121.92 | 79.82 | 79.71-79.92 |
| Others | 25096 | 24.6 | 122.35 | 122.18-122.52 | 79.91 | 79.78-80.03 |
| **Behavioral and Physiological** | |  |  |  |  |  |
| **Waist Hip Ratio category** | |  |  |  |  |  |
| >/=0.90 | 44168 | 43.4 | 124.20 | 124.07-124.33 | 82.01 | 81.91-82.11 |
| <0.90 | 40523 | 39.8 | 119.73 | 119.61-119.86 | 77.67 | 77.57-77.76 |
| **History of Alcohol use** |  |  |  |  |  |  |
| No | 69279 | 68.0 | 121.31 | 121.21-121.41 | 79.12 | 79.04-79.19 |
| Yes | 20845 | 20.5 | 124.49 | 124.30-124.69 | 82.54 | 82.39-82.69 |
| **History of tobacco use** |  |  |  |  |  |  |
| No | 78259 | 76.8 | 121.92 | 121.82-122.02 | 79.74 | 79.67-79.81 |
| Yes | 11866 | 11.7 | 122.87 | 122.62-123.12 | 81.01 | 80.82-81.19 |
| **Frequency of fruit intake** |  |  |  |  |  |  |
| Never | 1490 | 1.5 | 121.10 | 120.37-121.83 | 80.12 | 79.58-80.67 |
| Daily | 10590 | 10.4 | 122.72 | 122.46-122.98 | 80.28 | 80.08-80.48 |
| Weekly | 39478 | 38.8 | 122.38 | 122.25-122.52 | 80.21 | 80.11-80.31 |
| Occasionally | 38567 | 37.9 | 121.55 | 121.41-121.68 | 79.49 | 79.39-79.59 |
| **Frequency of fried food intake** | |  |  |  |  |  |
| Never | 6883 | 6.8 | 122.53 | 122.19-122.86 | 80.68 | 80.44-80.93 |
| Daily | 8018 | 7.9 | 122.46 | 122.15-122.76 | 79.72 | 79.50-79.94 |
| Weekly | 32160 | 31.6 | 121.67 | 121.53-121.82 | 79.62 | 79.51-79.73 |
| Occasionally | 43063 | 42.3 | 122.17 | 122.04-122.30 | 80.04 | 79.94-80.14 |
| **Co-morbidities** |  |  |  |  |  |  |
| Hypertension | 3078 | 3.0 | 132.26 | 131.57-132.94 | 87.58 | 87.12-88.04 |
| Diabetes | 2497 | 2.5 | 127.16 | 126.54-127.78 | 84.24 | 83.82-84.66 |
| Cardiovascular disease | 956 | 0.9 | 122.92 | 121.98-123.86 | 81.41 | 80.76-82.05 |

*Includes Sikh, Buddhist, Jain, Jewish and others

Table 2 and Table 4 highlight the key findings from adult female population. The systolic and diastolic blood pressure of women increases with increasing age as shown in Figure 3 and Figure 4, which is also evident from the statistically significant trends in the increasing odds ratio. Though DBP does not have any association with marital status, the SBP is showing a significant association with marital status. Married women are having 21% reduced risk of developing raised SBP compared to never married women. Muslim women are 1.13 and 1.16times increased risk of developing raised SBP and DBP respectively, compared Hindu women. Others category (Sikh, Buddhist, Jain and Jewish) women are having 1.45 and 1.32 times higher risk of developing raised SBP and DBP respectively, compared to Hindu women. With an increase in educational status, the odds of developing raised SBP and DBP decreases. When compared to no education women, the primary, secondary and higher educated women show 9%, 8% and 28% reduced risk having higher SBP. The higher educated women show 20%reduced risk having higher DBP in comparison with no education women. Middle, richer and richest quintile shows 1.13, 1.25 and 1.23 times of higher risk of developing raised DBP compared to poorest quintile. However, there was no association observed between SBP and wealth index among women. The employed women are 14% and 5% lesser risk of developing raised SBP and DBP respectively, compared to unemployed women. There was no association observed between raised BP and residence among women. Women belonging to central, north-east and south zone have 1.29, 1.28 and 1.20 times increasedrisk of developing raised SBP respectively, compared to women from north zone. Similarly, women belonging to central, north-east and south zone have 1.22, 1.17 and 1.32 times increased risk of developing raised DBP respectively. Women belonging to ST caste have 1.27 and 1.16 times higher risk developing raised SBP and DBP respectively, whereas women belonging to OBC have8% lesser risk of developing raised DBP.

**Figure 3: Female systolic blood pressure across different age group in the study population**

# Figure 4: Female diastolic blood pressure across different age group in the study population

The women having WHR more than 0.85 have 1.38 and 1.38 times higher risk of developing raised SBP and DBP respectively. Women having a history of alcohol consumption have 1.44 and 1.56 times higher risk of developing raised SBP and DBP respectively. There was no association observed between raised BP and tobacco use among women. There was no association observed between raised BP and fruit intake among women. Regarding fruit intake, daily, weekly and occasionally consuming men have 20%. 9% and 15% reduced risk of developing raised DBP respectively, compared to never category men. Regarding fried food intake, weekly and occasional consuming women have 24% and 19% reduced risk of developing raised SBP, compared to never category women. Daily, weekly and occasional consumers of fried food have 13%, 17% and 14% reduced risk of developing raised DBP respectively, compared to never category women. Women with diabetes have 1.53 and 1.60 times increased risk of developing raised SBP and DBP respectively.

# Table 2: Distribution of systolic and diastolic blood pressure among females according to sociodemographic, behavioral and physiological characteristics of the study population, NFHS-5, 2019-21, India (N=7,24,115)

| **Characteristics** | **N** | **(%)** | **Systolic blood pressure** | | **Diastolic blood pressure** | |
| --- | --- | --- | --- | --- | --- | --- |
|  |  |  | **Weighted mean** | **95%CI** | **Weighted mean** | **95%CI** |
| **Socio-demography** |  |  |  |  |  |  |
| **Age category (in years)** |  |  |  |  |  |  |
| 15-19 | 111820 | 15.4 | 109.47 | 109.40-109.53 | 72.34 | 72.29-72.39 |
| 20-24 | 109961 | 15.2 | 110.66 | 110.59-110.72 | 73.99 | 73.94-74.04 |
| 25-29 | 108578 | 15.0 | 112.42 | 112.35-112.49 | 75.84 | 75.79-75.89 |
| 30-34 | 93505 | 12.9 | 115.09 | 115.01-115.17 | 77.93 | 77.87-77.98 |
| 35-39 | 90926 | 12.6 | 117.86 | 117.77-117.95 | 79.68 | 79.62-79.75 |
| 40-44 | 76627 | 10.6 | 121.43 | 121.32-121.53 | 81.25 | 81.18-81.32 |
| 45-49 | 79726 | 11.0 | 124.71 | 124.59-124.83 | 82.27 | 82.20-82.34 |
| **Marital status** |  |  |  |  |  |  |
| Never married | 155694 | 21.5 | 110.37 | 110.31-110.42 | 73.46 | 73.42-73.50 |
| Currently married | 486713 | 67.2 | 116.51 | 116.47-116.55 | 78.14 | 78.11-78.16 |
| Widowed /Separated  /Divorced | 28736 | 4.0 | 120.02 | 119.83-120.21 | 80.42 | 80.30-80.54 |
| **Religion** |  |  |  |  |  |  |
| Hindu | 550204 | 76.0 | 115.09 | 115.05-115.13 | 77.08 | 77.06-77.11 |
| Muslim | 86887 | 12.0 | 115.80 | 115.71-115.90 | 77.28 | 77.21-77.34 |
| Christian | 15773 | 2.2 | 114.55 | 114.31-114.79 | 77.26 | 77.09-77.41 |
| Others* | 18280 | 2.5 | 117.60 | 117.39-117.82 | 78.46 | 78.32-78.61 |
| **Educational status** |  |  |  |  |  |  |
| No education | 152941 | 21.1 | 118.82 | 118.74-118.89 | 79.32 | 79.27-79.37 |
| Primary | 79627 | 11.0 | 117.47 | 117.37-117.57 | 78.68 | 78.62-78.75 |
| Secondary | 336361 | 46.5 | 113.84 | 113.79-113.88 | 76.18 | 76.14-76.21 |
| Higher | 102214 | 14.1 | 112.74 | 112.66-112.82 | 75.91 | 75.85-75.96 |
| **Wealth Index of the household** | |  |  |  |  |  |
| Poorest (I quintile) | 125420 | 17.3 | 115.07 | 114.99-115.14 | 76.55 | 76.50-76.61 |
| Poorer (II quintile) | 135405 | 18.7 | 114.83 | 114.76-114.91 | 76.76 | 76.71-76.81 |

| Middle (III quintile) | 139011 | 19.2 | 114.99 | 114.92-115.06 | 77.15 | 77.10-77.20 |
| --- | --- | --- | --- | --- | --- | --- |
| Richer (IV quintile) | 140220 | 19.4 | 115.30 | 115.22-115.37 | 77.45 | 77.40-77.50 |
| Richest (V quintile) | 131087 | 18.1 | 116.01 | 115.94-116.09 | 77.80 | 77.75-77.85 |
| **Current occupation** |  |  |  |  |  |  |
| Unemployed | 74844 | 10.3 | 114.77 | 114.67-114.87 | 76.82 | 76.75-76.89 |
| Employed | 25627 | 3.5 | 116.20 | 116.02-116.37 | 78.11 | 77.99-78.22 |
| **Residence** |  |  |  |  |  |  |
| Urban | 212706 | 29.4 | 115.44 | 115.38-115.50 | 77.52 | 77.48-77.56 |
| Rural | 458437 | 63.3 | 115.14 | 115.10-115.18 | 76.97 | 76.95-77.00 |
| **Region** |  |  |  |  |  |  |
| North | 95548 | 13.2 | 117.40 | 117.31-117.48 | 78.04 | 77.98-78.09 |
| Central | 168057 | 23.2 | 116.32 | 116.25-116.38 | 77.54 | 77.49-77.58 |
| East | 152178 | 21.0 | 114.33 | 114.26-114.40 | 76.40 | 76.36-76.45 |
| Northeast | 24576 | 3.4 | 116.89 | 116.71-117.07 | 77.62 | 77.50-77.74 |
| West | 93541 | 12.9 | 114.44 | 114.35-114.53 | 76.88 | 76.82-76.94 |
| South | 137243 | 19.0 | 113.65 | 113.58-113.73 | 76.98 | 76.93-77.03 |
| **Caste** |  |  |  |  |  |  |
| SC | 148158 | 20.5 | 114.53 | 114.46-114.60 | 76.87 | 76.82-76.92 |
| ST | 63553 | 8.8 | 116.57 | 116.47-116.68 | 77.57 | 77.50-77.65 |
| OBC | 290700 | 40.1 | 114.94 | 114.89-114.99 | 76.98 | 76.95-77.02 |
| Others | 168734 | 23.3 | 115.86 | 115.80-115.93 | 77.52 | 77.47-77.56 |
| **Behavioral and physiological** | |  |  |  |  |  |
| **Waist Hip Ratio category** | |  |  |  |  |  |
| >/=0.85 | 382488 | 52.8 | 116.87 | 116.82-116.91 | 78.06 | 78.03-78.09 |
| <0.85 | 257040 | 35.5 | 112.80 | 112.75-112.85 | 75.78 | 75.75-75.82 |
| **History of Alcohol use** |  |  |  |  |  |  |
| No | 666052 | 92.0 | 115.20 | 115.17-115.24 | 77.12 | 77.10-77.15 |
| Yes | 5092 | 0.7 | 119.76 | 119.32-120.21 | 80.38 | 80.09-80.68 |
| **History of tobacco use** |  |  |  |  |  |  |
| No | 670500 | 92.6 | 115.24 | 115.20-115.27 | 77.15 | 77.12-77.17 |
| Yes | 644 | 0.1 | 115.62 | 114.43-116.80 | 77.57 | 76.83-78.31 |
| **Frequency of fruit intake** | |  |  |  |  |  |
| Never | 10901 | 1.5 | 115.49 | 115.22-115.77 | 77.44 | 77.25-77.62 |
| Daily | 81322 | 11.2 | 114.68 | 114.59-114.78 | 76.95 | 76.89-77.02 |
| Weekly | 247699 | 34.2 | 115.23 | 115.17-115.28 | 77.20 | 77.16-77.24 |
| Occasionally | 331221 | 45.7 | 115.37 | 115.32-115.42 | 77.15 | 77.12-77.18 |
| **Frequency of fried food intake** | |  |  |  |  |  |
| Never | 29516 | 4.1 | 116.03 | 115.86-116.20 | 77.79 | 77.67-77.90 |
| Daily | 49013 | 6.8 | 115.10 | 114.97-115.23 | 77.05 | 76.96-77.13 |
| Weekly | 237557 | 32.8 | 115.13 | 115.07-115.19 | 77.05 | 77.01-77.09 |
| Occasionally | 355057 | 49.0 | 115.26 | 115.22-115.31 | 77.17 | 77.14-77.21 |
| **Co-morbidities** |  |  |  |  |  |  |
| Hypertension | 32171 | 4.4 | 124.18 | 123.97-124.40 | 82.96 | 82.83-83.10 |
| Diabetes | 12725 | 1.8 | 123.84 | 123.54-124.14 | 82.51 | 82.34-82.69 |
| Cardiovascular disease | 4871 | 0.7 | 117.99 | 117.54-118.43 | 79.24 | 78.95-79.53 |

*Includes Sikh, Buddhist, Jain, Jewish and others

# Table 3: Multivariate logistic regression analysis to determine the factors associated with elevated systolic and diastolic blood pressure among male, NFHS-5, 2019-21, India

|  | **SBP (>/=140mmHg)** | | | **DBP (>/=90mmHg)** | | |
| --- | --- | --- | --- | --- | --- | --- |
| **Characteristics** | **OR** | **95% CI** | | **OR** | **95% CI** | |
|  |  | **Lowe r** | **Uppe r** |  | **Lowe r** | **Uppe r** |
| **Socio-demographic characteristics** |  |  |  |  |  |  |
| **Age category (in years)** |  |  |  |  |  |  |
| 15-19 | 1 |  |  | 1 |  |  |
| 20-24 | 2.64* | 2.22 | 3.14 | 2.14* | 1.86 | 2.46 |
| 25-29 | 3.13* | 2.62 | 3.75 | 2.98* | 2.58 | 3.43 |
| 30-34 | 4.23* | 3.51 | 5.10 | 4.83* | 4.18 | 5.58 |
| 35-39 | 5.99* | 4.97 | 7.22 | 6.50* | 5.62 | 7.53 |
| 40-44 | 9.49* | 7.88 | 11.4 2 | 8.74* | 7.55 | 10.1 3 |
| 45-49 | 12.51 * | 10.4 1 | 15.0 4 | 9.76* | 8.43 | 11.3 0 |
| 50-54 | 17.29 * | 14.3 6 | 20.8 1 | 10.56 * | 9.10 | 12.2 5 |
| **Marital status** |  |  |  |  |  |  |
| Never married | 1 |  |  | 1 |  |  |
| Currently married | 0.95 | 0.86 | 1.05 | 1.12* | 1.04 | 1.21 |
| Widowed /Separated /Divorced | 1.09 | 0.90 | 1.32 | 1.15 | 0.98 | 1.36 |
| **Religion** |  |  |  |  |  |  |
| Hindu | 1 |  |  | 1 |  |  |
| Muslim | 0.90* | 0.83 | 0.98 | 0.86* | 0.81 | 0.92 |
| Christian | 0.92 | 0.79 | 1.06 | 0.89* | 0.78 | 1.00 |
| Others* | 1.29* | 1.11 | 1.49 | 1.00 | 0.88 | 1.13 |
| **Educational status** |  |  |  |  |  |  |
| No education | 1 |  |  | 1 |  |  |
| Primary | 1.14* | 1.04 | 1.25 | 1.09* | 1.01 | 1.18 |
| Secondary | 1.20* | 1.11 | 1.30 | 1.24* | 1.16 | 1.33 |
| Higher | 1.01 | 0.91 | 1.12 | 1.12* | 1.03 | 1.21 |
| **Wealth Index of the household** |  |  |  |  |  |  |
| Poorest (I quintile) | 1 |  |  | 1 |  |  |
| Poorer (II quintile) | 1.01 | 0.93 | 1.11 | 1.17* | 1.08 | 1.26 |
| Middle (III quintile) | 1.07 | 0.97 | 1.18 | 1.32* | 1.22 | 1.43 |
| Richer (IV quintile) | 1.12* | 1.01 | 1.23 | 1.32* | 1.22 | 1.43 |
| Richest (V quintile) | 1.14* | 1.02 | 1.28 | 1.35* | 1.23 | 1.49 |
| **Current occupation** |  |  |  |  |  |  |
| Unemployed | 1 |  |  | 1 |  |  |
| Employed | 0.95 | 0.88 | 1.03 | 0.97 | 0.91 | 1.04 |
| **Residence** |  |  |  |  |  |  |
| Urban | 1 |  |  | 1 |  |  |
| Rural | 0.92* | 0.86 | 0.97 | 0.92* | 0.88 | 0.97 |
| **Region** |  |  |  |  |  |  |
| North | 1 |  |  | 1 |  |  |
| Central | 1.05 | 0.93 | 1.17 | 1.06 | 0.97 | 1.17 |
| East | 0.66* | 0.59 | 0.73 | 0.67* | 0.62 | 0.74 |
| Northeast | 1.01 | 0.88 | 1.16 | 0.97 | 0.86 | 1.09 |
| West | 0.75* | 0.68 | 0.83 | 0.95 | 0.88 | 1.04 |
| South | 1.03 | 0.93 | 1.13 | 1.27* | 1.17 | 1.38 |
| **Caste** |  |  |  |  |  |  |
| SC | 1 |  |  | 1 |  |  |
| ST | 1.15* | 1.04 | 1.28 | 1.04 | 0.95 | 1.12 |
| OBC | 0.95 | 0.89 | 1.02 | 0.89* | 0.84 | 0.94 |
| Others | 1.01 | 0.93 | 1.09 | 0.91* | 0.85 | 0.97 |
| **Behavioral and physiological characteristics** |  |  |  |  |  |  |
| **Waist Hip Ratio (WHR) category** |  |  |  |  |  |  |
| <0.90 | 1 |  |  | 1 |  |  |
| >/=0.90 | 1.46* | 1.39 | 1.54 | 1.62* | 1.55 | 1.69 |
| **History of Alcohol use** |  |  |  |  |  |  |
| No | 1 |  |  | 1 |  |  |
| Yes | 1.28* | 1.20 | 1.35 | 1.50* | 1.43 | 1.57 |
| **History of tobacco use** |  |  |  |  |  |  |
| No | 1 |  |  | 1 |  |  |
| Yes | 1.20* | 1.11 | 1.29 | 1.05 | 0.98 | 1.11 |
| **Frequency of fruit intake** |  |  |  |  |  |  |
| Never | 1 |  |  | 1 |  |  |
| Daily | 1.15 | 0.93 | 1.42 | 0.80* | 0.68 | 0.95 |
| Weekly | 1.09 | 0.89 | 1.33 | 0.91 | 0.78 | 1.06 |
| Occasionally | 1.05 | 0.86 | 1.29 | 0.85* | 0.73 | 0.99 |
| **Frequency of fried food intake** |  |  |  |  |  |  |
| Never | 1 |  |  | 1 |  |  |
| Daily | 1.18* | 1.04 | 1.33 | 0.90* | 0.81 | 0.99 |
| Weekly | 0.99 | 0.89 | 1.09 | 0.91* | 0.84 | 0.98 |
| Occasionally | 1.08 | 0.99 | 1.19 | 0.93* | 0.86 | 1.00 |
| **Co-morbidities** |  |  |  |  |  |  |
| Diabetes | 1.12 | 1.00 | 1.25 | 1.01 | 0.92 | 1.12 |
| Cardiovascular disease | 0.81 | 0.65 | 1.00 | 0.84 | 0.70 | 1.01 |

# *p value<0.05 Table 4: Multivariate logistic regression analysis to determine the factors associated with elevated systolic and diastolic blood pressure among female, NFHS-5, 2019-21, India

|  | **SBP (>/=140mmHg)** | | | **DBP (>/=90mmHg)** | | |
| --- | --- | --- | --- | --- | --- | --- |
| **Characteristics** | **OR** | **95% CI** | | **OR** | **95% CI** | |
|  |  | **Lower** | **Upper** |  | **Lower** | **Upper** |
| **Socio-demographic characteristics** |  |  |  |  |  |  |
| **Age category (in years)** |  |  |  |  |  |  |
| 15-19 | 1 |  |  | 1 |  |  |
| 20-24 | 1.88* | 1.40 | 2.52 | 1.71* | 1.45 | 2.01 |
| 25-29 | 3.89* | 2.91 | 5.21 | 2.49* | 2.10 | 2.95 |
| 30-34 | 7.25* | 5.43 | 9.68 | 4.10* | 3.46 | 4.86 |
| 35-39 | 12.76* | 9.60 | 16.96 | 6.77* | 5.73 | 8.00 |
| 40-44 | 24.53* | 18.48 | 32.55 | 9.01* | 7.61 | 10.66 |
| 45-49 | 37.07* | 27.97 | 49.13 | 11.16* | 9.44 | 13.20 |
| **Marital status** |  |  |  |  |  |  |
| Never married | 1 |  |  | 1 |  |  |
| Currently married | 0.79* | 0.66 | 0.95 | 1.05 | 0.93 | 1.19 |
| Widowed /Separated /Divorced | 0.95 | 0.77 | 1.18 | 1.12 | 0.96 | 1.30 |
| **Religion** |  |  |  |  |  |  |
| Hindu | 1 |  |  | 1 |  |  |
| Muslim | 1.13* | 1.03 | 1.25 | 1.16* | 1.08 | 1.25 |
| Christian | 0.90 | 0.74 | 1.11 | 0.97 | 0.84 | 1.13 |
| Others | 1.45* | 1.22 | 1.71 | 1.32* | 1.16 | 1.51 |
| **Educational status** |  |  |  |  |  |  |
| No education | 1 |  |  | 1 |  |  |
| Primary | 0.91 | 0.83 | 1.01 | 1.07 | 0.99 | 1.15 |
| Secondary | 0.92* | 0.84 | 1.00 | 0.99 | 0.93 | 1.06 |
| Higher | 0.72* | 0.62 | 0.82 | .80* | 0.72 | 0.88 |
| **Wealth Index of the household** |  |  |  |  |  |  |
| Poorest (I quintile) | 1 |  |  | 1 |  |  |
| Poorer (II quintile) | 0.96 | 0.86 | 1.06 | 1.08 | 0.99 | 1.17 |
| Middle (III quintile) | 1.07 | 0.96 | 1.20 | 1.13* | 1.04 | 1.23 |
| Richer (IV quintile) | 1.13 | 1.00 | 1.27 | 1.25* | 1.14 | 1.37 |
| Richest (V quintile) | 1.12 | 0.98 | 1.29 | 1.23* | 1.11 | 1.37 |
| **Current occupation** |  |  |  |  |  |  |
| Unemployed | 1 |  |  | 1 |  |  |
| Employed | 0.86* | 0.80 | 0.92 | 0.95 | 0.90 | 1.00 |
| **Residence** |  |  |  |  |  |  |
| Urban | 1 |  |  | 1 |  |  |
| Rural | 0.98 | 0.90 | 1.06 | 0.95 | 0.90 | 1.01 |
| **Region** |  |  |  |  |  |  |
| North | 1 |  |  | 1 |  |  |
| Central | 1.29* | 1.15 | 1.44 | 1.22* | 1.12 | 1.33 |
| East | 1.01 | 0.90 | 1.14 | 0.97 | 0.88 | 1.06 |
| Northeast | 1.28* | 1.07 | 1.54 | 1.17* | 1.01 | 1.35 |
| West | .94 | 0.83 | 1.07 | 1.07 | 0.98 | 1.17 |
| South | 1.20* | 1.07 | 1.34 | 1.32* | 1.21 | 1.43 |
| **Caste** |  |  |  |  |  |  |
| SC | 1 |  |  | 1 |  |  |
| ST | 1.27* | 1.12 | 1.44 | 1.16* | 1.06 | 1.28 |
| OBC | 0.96 | 0.88 | 1.04 | 0.92* | 0.86 | 0.98 |
| Others | 1.10 | 1.00 | 1.22 | 1.03 | 0.95 | 1.10 |
| **Behavioral and physiological characteristics** | |  |  |  |  |  |
| **Waist Hip Ratio (WHR) category** |  |  |  |  |  |  |
| <0.85 | 1 |  |  | 1 |  |  |
| >/=0.85 | 1.38* | 1.28 | 1.48 | 1.38* | 1.31 | 1.46 |
| **History of Alcohol use** |  |  |  |  |  |  |
| No | 1 |  |  | 1 |  |  |
| Yes | 1.44* | 1.13 | 1.84 | 1.56* | 1.28 | 1.89 |
| **History of tobacco use** |  |  |  |  |  |  |
| No | 1 |  |  | 1 |  |  |
| Yes | 1.01 | 0.38 | 2.73 | 0.44 | 0.15 | 1.27 |
| **Frequency of fruit intake** |  |  |  |  |  |  |
| Never | 1 |  |  | 1 |  |  |
| Daily | 0.96 | 0.75 | 1.23 | 0.83 | 0.69 | 1.00 |
| Weekly | 1.00 | 0.79 | 1.26 | 0.88 | 0.74 | 1.05 |
| Occasionally | 0.93 | 0.74 | 1.18 | 0.85 | 0.72 | 1.02 |
| **Frequency of fried food intake** |  |  |  |  |  |  |
| Never | 1 |  |  | 1 |  |  |
| Daily | 0.85 | 0.71 | 1.01 | 0.87* | 0.75 | 0.99 |
| Weekly | 0.76* | 0.66 | 0.88 | 0.83* | 0.75 | 0.93 |
| Occasionally | 0.81* | 0.71 | 0.93 | 0.86* | 0.77 | 0.96 |
| **Co-morbidities** |  |  |  |  |  |  |
| Diabetes | 1.53* | 1.32 | 1.77 | 1.60* | 1.42 | 1.81 |
| Cardiovascular disease | 1.06 | 0.80 | 1.41 | 1.00 | 0.79 | 1.25 |

*p value<0.05

Table 5, Figure 5 and Figure 6 highlights the cut off age in years for adult men using Receiver Operating Characteristics (ROC) Curve. The ROC has been used to evaluate and compare the performance of age as the screening tool to identify the binary outcome variable (raised blood pressure). The Youden index was used as a summary measure of the ROC curve to select the optimal cut off point for the age within the age group. The ROC analysis yielded an AUC of 0.71 (95% CI: 0.70–0.72) for males and 0.68 (95% CI: 0.67–0.69) for females when using age to predict elevated systolic blood pressure (SBP). The 35 to 39 years age group has shown the least difference between sensitivity and specificity and hence it can be suggested as the ideal screening age among adult men for raised SBP and DBP. Table 6, Figure 7 and Figure 8 highlights the cut off age in years for adult women using ROC Curve. Similar to adult men, the 35 to 39 years age group has shown the least difference between sensitivity and specificity and hence it can be suggested as the ideal screening age among adult women for raised SBP and DBP. The Youden index identified 36.5 years as the age cutoff with the best balance of sensitivity (57.9%) and specificity (49.7%) for men, and similar findings for women, supporting the recommendation of 35 years as a screening initiation point **Figure 5: Male systolic blood pressure across different age group in the study population**

**Figure 6: Male diastolic blood pressure across different age group in the study population**

**Figure 7: Female systolic blood pressure across different age group in the study population**

**Figure 8: Female diastolic blood pressure across different age group in the study population**

# Table 5:Cut-off in years for males based on ROC curves

| **Age category (in years)** | **SBP (>/=140mmHg)** | | | **DBP (>/=90mmHg)** | | |
| --- | --- | --- | --- | --- | --- | --- |
|  | Cut off | Sensitivity | Specificity | Cut off | Sensitivity | Specificity |
| 15-19 | 16.5 | 70.0% | 41.8% | 17.5 | 52.6% | 61.4% |
| 20-24 | 21.5 | 63.2% | 41.1% | 21.5 | 66.5% | 41.3% |
| 25-29 | 26.5 | 60.1% | 43.2% | 26.5 | 61.2% | 43.5% |
| 30-34 | 31.5 | 60.6% | 44.4% | 31.5 | 57.9% | 44.4% |
| 35-39 | 36.5 | 57.9% | 49.7% | 36.5 | 56.5% | 50.2% |
| 40-44 | 41.5 | 57.0% | 48.2% | 40.5 | 70.3% | 33.3% |
| 45-49 | 46.5 | 57.1% | 49.5% | 46.5 | 55.0% | 49.4% |
| 50-54 | 51.5 | 58.1% | 44.5% | 50.5 | 74.6% | 27.1% |
| 15-54 (overall) | 35.5 | 68.0% | 63.9% | 30.5 | 78.8% | 52.3% |

# Table 6:Cut-off in years for females based on ROC curves

| **Age category (in years)** | **SBP (>/=140mmHg)** | | | **DBP (>/=90mmHg)** | | |
| --- | --- | --- | --- | --- | --- | --- |
|  | Cut off | Sensitivity | Specificity | Cut off | Sensitivity | Specificity |
| 15-19 | 16.5 | 66.3% | 40.4% | 16.5 | 64.2% | 40.4% |
| 20-24 | 21.5 | 64.1% | 40.4% | 21.5 | 65.4% | 40.6% |
| 25-29 | 26.5 | 63.1% | 43.7% | 26.5 | 62.6% | 43.9% |
| 30-34 | 31.5 | 63.1% | 44.5% | 31.5 | 61.1% | 44.7% |
| 35-39 | 36.5 | 60.6% | 47.4% | 36.5 | 57.8% | 47.6% |
| 40-44 | 41.5 | 59.4% | 46.2% | 41.5 | 57.5% | 46.2% |
| 45-49 | 46.5 | 59.3% | 46.4% | 46.5 | 56.9% | 46.1% |
| 15-49 (overall) | 34.5 | 78.2% | 65.4% | 32.5 | 72.5% | 61.5% |

Table 7 presents the diagnostic performance of age as a predictor of hypertension using ROC analysis for both systolic and diastolic blood pressure in males and females. The analysis identifies 36.5 years as the optimal cut-off age for initiating hypertension screening in both sexes, based on the Youden Index. Among males, the area under the curve (AUC) was 0.71 for systolic BP and 0.69 for diastolic BP, indicating fair discriminatory ability. In females, the AUC values were slightly lower at 0.68 for systolic BP and 0.66 for diastolic BP. Sensitivity and specificity values at this cut-off were reasonably balanced, ranging from 56% to 61% sensitivity and 47% to 50% specificity. These findings support 35–36 years as a data-driven and practical threshold for routine hypertension screening among Indian adults.

Table 7: ROC-Based Diagnostic Performance of Age for Predicting Hypertension in Indian Adults (NFHS-5, 2019–21)

| **Sex** | **Outcome** | **Cut-off**  **Age (years)** | **AUC (95%**  **CI)** | **Sensitivity (%)** | **Specificity (%)** | **Youden Index** |
| --- | --- | --- | --- | --- | --- | --- |
| **Male** | SBP ≥140 mmHg | 36.5 | 0.71 (0.70–  0.72) | 57.9 | 49.7 | 0.076 |
|  | DBP ≥90 mmHg | 36.5 | 0.69 (0.68–  0.70) | 56.5 | 50.2 | 0.067 |
| **Female** | SBP ≥140 mmHg | 36.5 | 0.68 (0.67–  0.69) | 60.6 | 47.4 | 0.08 |
|  | DBP ≥90 mmHg | 36.5 | 0.66 (0.65–  0.67) | 57.8 | 47.6 | 0.054 |

**Discussion:**

The present study utilized data from the NFHS-5 survey, which includes data from 1,01,839 men and 7,24,115 women participants. Hypertension, identified in 28.5% of Indian adults aged 18-69, remains a major public health challenge, contributing significantly to the rising burden of non-communicable diseases (NCDs) in India(19,20). The findings emphasize the need for targeted screening interventions, particularly focusing on desired age for screening, along with socio-demographic, behavioral, and physiological factors.

Comparison with Global and National Trends

Globally, the prevalence of hypertension among adults was 32% in women and 34% in men in 2019, with 41% of women and 51% of men being unaware of their hypertensive status (19). This study's findings align with global trends, showing a linear increase in blood pressure with age, emphasizing the need for timely detection. The study identifies the age group of 35-39 years as a critical point for initiating hypertension screening, suggesting that approximately 60% of adults in this age group have elevated blood pressure, a statistic that parallels the 60% sensitivity identified in the current study(21).

The World Health Organization (WHO) emphasizes opportunistic screening for all adults in primary care settings but does not recommend a specific age threshold(22). The US Preventive Services Task Force (USPSTF) recommends annual hypertension screening for adults aged ≥40 years or younger adults with risk factors, and every 3–5 years for those aged 18–39 years without risk factors(21). The Indian NPCDCS program currently recommends annual screening starting at 30 years of age(23), but this recommendation lacks age-stratified evidence from large-scale national datasets. Our study contributes by empirically identifying 36.5 years as the optimal cut-off based on ROC analysis, offering the first age-specific evidence from a nationally representative Indian sample (NFHS-5). Compared to prior Indian studies such as Prenissl et al. (2019), which highlighted awareness gaps but did not define an ideal screening age(24), our findings offer direct policy guidance.

In India, the National Family Health Survey (NFHS-5) data highlights a gap in hypertension awareness, with only 27% of hypertensive individuals aware of their condition, 14.5% receiving treatment, and just 12.6% having controlled blood pressure(20). These figures underscore the need for improved screening strategies. This study further suggests that starting screening at 35 years could potentially capture a significant portion of undiagnosed cases, contributing to the national goal of reducing hypertension prevalence by 25% by 2025 (25).This gap in awareness and management is concerning, especially considering the high prevalence of hypertension, which is a major risk factor for cardiovascular diseases, including stroke and heart attacks(26).

One of the critical challenges in managing hypertension in India is the low rate of awareness. Studies have shown that lack of awareness is often due to the asymptomatic nature of hypertension, where individuals do not experience symptoms until significant damage has occurred to vital organs (27). This underlines the importance of regular screening to identify individuals with elevated blood pressure before complications arise.

Moreover, the treatment rates for hypertension are alarmingly low. Even among those diagnosed, the rate of treatment is just 14.5%, indicating significant barriers to accessing healthcare, such as economic constraints, lack of healthcare infrastructure, and inadequate follow-up (28). Cultural beliefs and practices also play a role in influencing health-seeking behavior, with many individuals relying on traditional medicine or home remedies instead of seeking formal medical treatment(29).

The control rate, with only 12.6% of hypertensive individuals maintaining their blood pressure within recommended levels, highlights the challenges in ongoing management and adherence to treatment regimens(20). Poor adherence to prescribed medication, often due to side effects, lack of understanding of the chronic nature of hypertension, and the financial burden of long-term treatment, are significant factors contributing to this issue(30).

Given these challenges, the study suggests that initiating hypertension screening at age 35 could play a crucial role in capturing a significant portion of undiagnosed cases. The age group of 35-39 years is identified as a critical period where the prevalence of hypertension begins to rise sharply. Within this age group, the study found that 56.4% of males and 60.9% of females have elevated blood pressure, underscoring the importance of early detection(31).Early screening is essential because evidence shows that hypertension detected and managed early significantly reduces the risk of cardiovascular events later in life. For example, the Framingham Heart Study demonstrated that individuals who developed hypertension by their mid-30s had a markedly higher lifetime risk of cardiovascular disease, including heart failure, myocardial infarction, and stroke(32).

The benefits of early screening are further supported by findings from the SPRINT trial, which showed that intensive blood pressure management in individuals at risk significantly reduced the rates of cardiovascular events and all-cause mortality(33). Additionally, a systematic review of hypertension management strategies in low- and middle-income countries highlighted that early detection through community-based screening programs effectively reduces the burden of hypertension-related complications(34).

Socio-Demographic Determinants

This study reveals a significant association between marital status and diastolic blood pressure (DBP), with married men exhibiting a 12% increased risk of elevated DBP compared to their unmarried counterparts. This finding resonates with previous studies indicating that married individuals, particularly men, may experience higher blood pressure due to marital stress(35). However, this stress-related increase in DBP needs to be balanced against the generally recognized health benefits of marriage, such as better social support and health behaviors(36).

The study also highlights a 10% lower risk of hypertension among Muslims compared to Hindus. This difference could be attributed to religious practices such as fasting during Ramadan, which has been shown to have beneficial effects on cardiovascular health(31). Further, dietary habits prevalent in Muslim communities, such as lower consumption of alcohol and possibly higher consumption of whole grains and legumes, may contribute to this reduced risk (37).

Education and wealth index were also significant predictors of hypertension. The study found that individuals with no formal education had a 20% higher risk of hypertension compared to those with higher education. This is consistent with global data suggesting that lower educational attainment is associated with poorer health outcomes due to factors like reduced health literacy and access to healthcare services (38). Moreover, the wealth index showed a clear gradient, with individuals in the highest quintile being 35% more likely to have elevated DBP, possibly due to lifestyle factors such as higher dietary intake of fats and sugars(39).

Behavioral and Physiological Risk Factors

Behavioral risk factors, particularly alcohol consumption and obesity, showed strong associations with hypertension in this study. Men who consumed alcohol were 28% more likely to have elevated systolic blood pressure (SBP), while those with a high waist-hip ratio (WHR) had a 46% higher risk of elevated SBP and a 62% higher risk of elevated DBP (40). These findings are supported by global statistics that link excessive alcohol consumption to an increased risk of hypertension due to its effects on the cardiovascular system (41).

Obesity, as indicated by WHR, is a well-established risk factor for hypertension, with studies showing that a 5 kg/m² increase in body mass index (BMI) can increase the risk of hypertension by 49% (42). The study's findings that higher WHR correlates with a substantial increase in hypertension risk emphasize the need for weight management interventions as part of hypertension prevention strategies.

Interestingly, the study reports a protective effect of fried food consumption on DBP, particularly in women, with those consuming fried foods weekly having a 24% reduced risk of elevated SBP and a 17% reduced risk of elevated DBP. This finding is contrary to the general understanding that fried foods, typically high in trans fats and sodium, contribute to hypertension(43). This unexpected result suggests that other factors, such as the type of oils used or the presence of other healthy behaviors, might be influencing these outcomes. For instance, some traditional Indian cooking practices involve the use of oils rich in unsaturated fats, which might offset the negative effects of frying(44). Further research is needed to disentangle these associations and understand the complex dietary patterns at play.

The study also highlights a gender difference in the impact of fruit intake on blood pressure, with daily fruit consumption associated with a 20% reduced risk of elevated DBP in men, but not in women. This difference could be due to variations in the types of fruits consumed or the overall dietary context in which fruit is eaten. Fruits are rich in potassium, which has been shown to counteract the effects of sodium and lower blood pressure (45). However, gender differences in nutrient metabolism or hormone levels may modify this protective effect, warranting further investigation.

Implications for Public Health and Policy

In India, the Ministry of Health and Family Welfare (MoHFW) recommends hypertension screening starting at age 30 (25). However, this study suggests that screening should be intensified from age 35 to capture those at risk earlier, as evidence suggests that undiagnosed hypertension in this group leads to increased cardiovascular morbidity later in life (35). Studies have shown that hypertension in individuals aged 35-44 is associated with a significantly higher risk of cardiovascular events, including heart attacks and strokes, compared to those diagnosed later in life(31,36). Furthermore, data from the Framingham Heart Study indicate that individuals who develop hypertension by their mid-30s have a higher lifetime risk of cardiovascular disease, emphasizing the importance of early detection and management(37). Additional research from the INTERHEART study confirms that the early onset of hypertension, particularly before age 40, substantially increases the risk of myocardial infarction, making early and targeted screening interventions crucial (38).

Based on the identified ROC thresholds and age-specific risk profiles, we recommend revising India’s hypertension screening guidelines to initiate routine screening from 35 years onward for both men and women. This adjustment balances feasibility with early detection impact, especially considering that SBP begins to rise substantially from this age group onward.

The study's findings also highlight the need for integrated public health strategies that address the underlying socio-economic and behavioral determinants of hypertension. For instance, public health campaigns should promote lifestyle changes such as reducing alcohol intake and managing body weight, particularly among younger adults. Given that only 14.5% of hypertensive individuals in India are receiving treatment, there is a clear need for improving access to care and ensuring that screening programs are effectively linked to treatment and follow-up services(20). Moreover, educational interventions aimed at improving health literacy could play a crucial role in reducing hypertension prevalence. The study's findings that lower educational attainment and wealth are associated with higher hypertension risk underscore the importance of addressing these social determinants of health through targeted public health interventions (46).

Limitations and Future Research

While this study provides valuable insights, it has limitations that should be acknowledged. The cross-sectional design limits the ability to establish causality between the identified risk factors and hypertension. Future research should focus on longitudinal studies to better understand the temporal relationships and causality (47). Additionally, the reliance on selfreported data for alcohol and tobacco use may introduce recall bias, potentially leading to underestimation of these behaviors' impact on hypertension (48). We acknowledge the possibility of regional disparities in healthcare access, awareness, and hypertension screening practices across different states and zones in India, which may influence screening rates and outcomes. This warrants further subnational analysis.

The study also did not include detailed dietary assessments, particularly sodium intake, which is a key contributor to hypertension. The average sodium intake in India is estimated to be over 10 grams per day, which is more than double the World Health Organization's recommended limit of 5 grams per day(49). Future studies should incorporate detailed dietary data, including sodium and potassium intake, to better understand their roles in hypertension in the Indian context (50).

**Conclusion:**

This study, based on nationally representative NFHS-5 data, provides robust evidence supporting the initiation of routine hypertension screening at 35 years of age for both men and women in India. Using ROC analysis, we identified 36.5 years as the optimal age cutoff with balanced sensitivity and specificity, suggesting a practical and data-driven revision to current national screening guidelines. While the cross-sectional nature of the dataset limits causal inferences, our findings warrant validation through longitudinal studies to confirm the predictive value of this threshold over time. To improve screening uptake among younger adults—especially in underserved regions—targeted community awareness campaigns, workplace-based screening programs, and mobile health interventions integrated with frontline health worker outreach (e.g., ASHAs) should be prioritized. These strategies can facilitate earlier diagnosis, improve blood pressure control, and reduce the burden of cardiovascular disease across the adult population.

Acknowledgement: None

Conflict of interest: None

**Data Availability Statement-**

Data is publicly available on demand with due permission from Demographic and Health

Survey (DHS).

**Reference:**

1. Zhou B, Carrillo-Larco RM, Danaei G, Riley LM, Paciorek CJ, Stevens GA, et al. Worldwide trends in hypertension prevalence and progress in treatment and control from 1990 to 2019: a pooled analysis of 1201 population-representative studies with 104 million participants. The Lancet. 2021 Sep 11;398(10304):957–80.
2. Amarchand R, Kulothungan V, Krishnan A, Mathur P. Hypertension treatment cascade in India: results from National Noncommunicable Disease Monitoring Survey. J Hum Hypertens. 2022 May 5;1–11.
3. Murray CJL, Aravkin AY, Zheng P, Abbafati C, Abbas KM, Abbasi-Kangevari M, et al. Global burden of 87 risk factors in 204 countries and territories, 1990–2019: a systematic analysis for the Global Burden of Disease Study 2019. The Lancet. 2020 Oct 17;396(10258):1223–49.
4. Unger T, Borghi C, Charchar F, Khan NA, Poulter NR, Prabhakaran D, et al. 2020 International Society of Hypertension Global Hypertension Practice Guidelines. Hypertension. 2020 Jun;75(6):1334–57.
5. US Preventive Services Task Force. Screening for Hypertension in Adults: US Preventive Services Task Force Reaffirmation Recommendation Statement. JAMA. 2021 Apr 27;325(16):1650–6.
6. Dhungana RR, Pedisic Z, Dhimal M, Bista B, de Courten M. Hypertension screening, awareness, treatment, and control: a study of their prevalence and associated factors in a nationally representative sample from Nepal. Glob Health Action. 2022 Dec 31;15(1):2000092.
7. Hypertension_full.pdf [Internet]. [cited 2022 Aug 4]. Available from: https://nhm.gov.in/images/pdf/guidelines/nrhm-guidelines/stg/Hypertension_full.pdf
8. Module for Multi-Purpose Workers - Prevention, Screening and Control of Common NCDS_2.pdf [Internet]. [cited 2022 Aug 5]. Available from:

https://main.mohfw.gov.in/sites/default/files/Module%20for%20Multi-Purpose%20Workers%20-

%20Prevention%2C%20Screening%20and%20Control%20of%20Common%20NCDS_2.pdf

9. Raghuveer P, Anand T, Tripathy JP, Nirgude AS, Reddy MM, Nandy S, et al. Opportunistic screening for diabetes mellitus and hypertension in primary care settings in Karnataka, India: a few steps forward but still some way to go. F1000Research. 2020 May 6;9:335.
10. Prenissl J, Manne-Goehler J, Jaacks LM, Prabhakaran D, Awasthi A, Bischops AC, et al. Hypertension screening, awareness, treatment, and control in India: A nationally representative crosssectional study among individuals aged 15 to 49 years. PLOS Med. 2019 May 3;16(5):e1002801.
11. Babu V, Sahu SK, Kanungo S. Hypertension control status and quality of care for hypertension among patients availing treatment from private sector: A cross-sectional study in urban field practice area of JIPMER, Puducherry. J Fam Med Prim Care. 2019 Jan;8(1):72–6.
12. Dhungana RR, Pedisic Z, Dhimal M, Bista B, de Courten M. Hypertension screening, awareness, treatment, and control: a study of their prevalence and associated factors in a nationally representative sample from Nepal. Glob Health Action. 2022 Dec 31;15(1):2000092.
13. Diederichs C, Neuhauser H. The frequency and determinants of blood pressure measurement by a health professional in Germany: A cross-sectional study. Medicine (Baltimore). 2019 Apr;98(16):e15093.
14. Amankwah E, Campbell NRC, Maxwell C, Onysko J, Quan H. Why Some Adult Canadians Do Not Have Blood Pressure Measured. J Clin Hypertens. 2007 Dec 19;9(12):944–51.
15. Lin JD, Chen YL, Wu CZ, Hsieh CH, Pei D, Liang YJ, et al. Identification of Normal Blood Pressure in Different Age Group. Medicine (Baltimore). 2016 Apr 8;95(14):e3188.
16. National Multisectoral Action Plan (NMAP) for Prevention and Control of Common NCDs (2017-22)_1.pdf [Internet]. [cited 2022 Aug 5]. Available from:

https://main.mohfw.gov.in/sites/default/files/National%20Multisectoral%20Action%20Plan%20%28N

MAP%29%20for%20Prevention%20and%20Control%20of%20Common%20NCDs%20%28201722%29_1.pdf

17. Hypertension [Internet]. [cited 2024 Aug 7]. Available from: https://www.who.int/newsroom/fact-sheets/detail/hypertension
18. World Health Organization. Waist circumference and waist-hip ratio : report of a WHO expert consultation, Geneva, 8-11 December 2008. 2011 [cited 2024 Aug 7]; Available from: https://iris.who.int/handle/10665/44583
19. Zhou B, Carrillo-Larco RM, Danaei G, Riley LM, Paciorek CJ, Stevens GA, et al. Worldwide trends in hypertension prevalence and progress in treatment and control from 1990 to 2019: a pooled analysis of 1201 population-representative studies with 104 million participants. The Lancet. 2021 Sep 11;398(10304):957–80.
20. Amarchand R, Kulothungan V, Krishnan A, Mathur P. Hypertension treatment cascade in India: results from National Noncommunicable Disease Monitoring Survey. J Hum Hypertens. 2022 May 5;1–11.
21. US Preventive Services Task Force. Screening for Hypertension in Adults: US Preventive Services Task Force Reaffirmation Recommendation Statement. JAMA. 2021 Apr 27;325(16):1650–6.
22. HEARTS: Technical package for cardiovascular disease management in primary health care:

Risk-based CVD management [Internet]. [cited 2025 May 16]. Available from:

https://www.who.int/publications/i/item/9789240001367

23. National Programme for Prevention and Control of Non Communicable Diseases (NP-NCD) Guidelines | Ministry of Health and Family Welfare | GOI [Internet]. [cited 2025 May 16]. Available from: https://mohfw.gov.in/?q=Major-Programmes/non-communicable-diseases-injury-trauma/Non-

Communicable-Disease-II/National-Programme-for-Prevention-and-Control-of-Cancer-Diabetes-

Cardiovascular-diseases-and-Stroke-NPCDCS

24. Prenissl J, Manne-Goehler J, Jaacks LM, Prabhakaran D, Awasthi A, Bischops AC, et al. Hypertension screening, awareness, treatment, and control in India: A nationally representative crosssectional study among individuals aged 15 to 49 years. PLoS Med. 2019 May;16(5):e1002801.
25. National Multisectoral Action Plan (NMAP) for Prevention and Control of Common NCDs (2017-22)_1.pdf [Internet]. [cited 2024 Sep 3]. Available from:

https://mohfw.gov.in/sites/default/files/National%20Multisectoral%20Action%20Plan%20%28NMAP

%29%20for%20Prevention%20and%20Control%20of%20Common%20NCDs%20%28201722%29_1.pdf

26. Koeppel L, Dittrich S, Miguel SB, Carmona S, Ongarello S, Vetter B, et al. Addressing the diagnostic gap in hypertension through possible interventions and scale-up: A microsimulation study.

PLOS Med [Internet]. 2022 Dec [cited 2024 Sep 4];19(12). Available from:

https://www.ncbi.nlm.nih.gov/pmc/articles/PMC9725126/

27. Awareness, Treatment, and Control of Hypertension among the Adult Population in Burkina Faso: Evidence from a Nationwide Population-Based Survey - PMC [Internet]. [cited 2024 Sep 4].

Available from: https://www.ncbi.nlm.nih.gov/pmc/articles/PMC8494593/

28. Anchala R, Kannuri NK, Pant H, Khan H, Franco OH, Di Angelantonio E, et al. Hypertension in India: a systematic review and meta-analysis of prevalence, awareness, and control of hypertension. J Hypertens. 2014 Jun;32(6):1170–7.
29. FR375.pdf [Internet]. [cited 2024 Sep 4]. Available from: https://dhsprogram.com/pubs/pdf/FR375/FR375.pdf
30. Residual lifetime risk for developing hypertension in middle-aged women and men: The Framingham Heart Study - PubMed [Internet]. [cited 2024 Sep 3]. Available from:

https://pubmed.ncbi.nlm.nih.gov/11866648/

31. A Randomized Trial of Intensive versus Standard Blood-Pressure Control | New England Journal of Medicine [Internet]. [cited 2024 Sep 3]. Available from:

https://www.nejm.org/doi/full/10.1056/NEJMoa1511939

32. Global Disparities of Hypertension Prevalence and Control: A Systematic Analysis of Population-based Studies from 90 Countries - PMC [Internet]. [cited 2024 Sep 4]. Available from:

https://www.ncbi.nlm.nih.gov/pmc/articles/PMC4979614/

33. National Health Policy 2017 (English) .pdf [Internet]. [cited 2024 Sep 4]. Available from:

https://nhsrcindia.org/sites/default/files/2021-

07/National%20Health%20Policy%202017%20%28English%29%20.pdf

34. Global action plan for the prevention and control of noncommunicable diseases 2013-2020 [Internet]. [cited 2024 Sep 4]. Available from: https://www.who.int/publications/i/item/9789241506236
35. Franklin SS, Gustin W, Wong ND, Larson MG, Weber MA, Kannel WB, et al. Hemodynamic patterns of age-related changes in blood pressure. The Framingham Heart Study. Circulation. 1997 Jul 1;96(1):308–15.
36. Yano Y, Reis JP, Colangelo LA, Shimbo D, Viera AJ, Allen NB, et al. Association of Blood

Pressure Classification in Young Adults Using the 2017 American College of Cardiology/American Heart Association Blood Pressure Guideline With Cardiovascular Events Later in Life. JAMA. 2018 Nov 6;320(17):1774–82.

37. Vasan RS, Beiser A, Seshadri S, Larson MG, Kannel WB, D’Agostino RB, et al. Residual lifetime risk for developing hypertension in middle-aged women and men: The Framingham Heart Study. JAMA. 2002 Feb 27;287(8):1003–10.
38. Yusuf S, Hawken S, Ounpuu S, Dans T, Avezum A, Lanas F, et al. Effect of potentially modifiable risk factors associated with myocardial infarction in 52 countries (the INTERHEART study): case-control study. Lancet Lond Engl. 2004 Sep 11;364(9438):937–52.
39. Popkin BM, Adair LS, Ng SW. Global nutrition transition and the pandemic of obesity in developing countries. Nutr Rev. 2012 Jan;70(1):3–21.
40. Cushman WC. Alcohol Consumption and Hypertension. J Clin Hypertens. 2007 May 31;3(3):166–70.
41. Diederichs C, Neuhauser H. The frequency and determinants of blood pressure measurement by a health professional in Germany: A cross-sectional study. Medicine (Baltimore). 2019 Apr;98(16):e15093.
42. Obesity Phenotypes, Diabetes, and Cardiovascular Diseases - PubMed [Internet]. [cited 2024 Sep 3]. Available from: https://pubmed.ncbi.nlm.nih.gov/32437302/
43. Bechthold A, Boeing H, Schwedhelm C, Hoffmann G, Knüppel S, Iqbal K, et al. Food groups and risk of coronary heart disease, stroke and heart failure: A systematic review and dose-response meta-analysis of prospective studies. Crit Rev Food Sci Nutr. 2017 Oct 17;59:00–00.
44. A clinical trial of the effects of dietary patterns on blood pressure. DASH Collaborative Research Group - PubMed [Internet]. [cited 2024 Sep 3]. Available from:

https://pubmed.ncbi.nlm.nih.gov/9099655/

45. Global Burden of Cardiovascular Diseases and Risk Factors, 1990-2019: Update From the GBD 2019 Study - PubMed [Internet]. [cited 2024 Sep 3]. Available from: https://pubmed.ncbi.nlm.nih.gov/33309175/
46. Menke A, Casagrande S, Geiss L, Cowie CC. Prevalence of and Trends in Diabetes Among Adults in the United States, 1988-2012. JAMA. 2015 Sep 8;314(10):1021–9.
47. Lin JD, Chen YL, Wu CZ, Hsieh CH, Pei D, Liang YJ, et al. Identification of Normal Blood Pressure in Different Age Group. Medicine (Baltimore). 2016 Apr 8;95(14):e3188.
48. Salt intake, stroke, and cardiovascular disease: meta-analysis of prospective studies | The BMJ

[Internet]. [cited 2024 Sep 3]. Available from: https://www.bmj.com/content/339/bmj.b4567

49. Powles J, Fahimi S, Micha R, Khatibzadeh S, Shi P, Ezzati M, et al. Global, regional and national sodium intakes in 1990 and 2010: A systematic analysis of 24 h urinary sodium excretion and dietary surveys worldwide. BMJ Open. 2013 Dec 20;3:e003733.
50. Mente A, O’Donnell M, Rangarajan S, Dagenais G, Lear S, McQueen M, et al. Associations of urinary sodium excretion with cardiovascular events in individuals with and without hypertension: a pooled analysis of data from four studies. Lancet Lond Engl. 2016 Jul 30;388(10043):465–75.

[Click here to access/download;Figure;figure 1.tif](https://www2.cloud.editorialmanager.com/pone/download.aspx?id=40624864&guid=ea9c4758-9e9c-4cd8-a80c-1f36cc6cb6ee&scheme=1)

Revised Manuscript with Track Changes

# Title: Hypertension Screening across Different Age Groups in Indian Adults: Evidence from Nationally Representative Cross-Sectional Data

Dr Prakash Mathiyalagen^1^, Dr Anand Rajagopal ^2^, Dr Kavita Vasudevan^3^, Dr Sridevi Gnanasekaran^4,^Dr Sharath BN^5^, Dr Jayanta Bora ^6^, Dr Sonu Goel ^7&^

^1^Associate Professor, Dept of Community Medicine, IGMC&RI, Puducherry

^2^Assistant Professor, Dept of Community Medicine, IGMC&RI, Puducherry

^3^Professor & Head, Dept of Community Medicine, IGMC&RI, Puducherry

^4^Senior Resident, Dept of Community Medicine, IGMC&RI, Puducherry

^5^Faculty, ESIC Medical College, Bengaluru

^6^Founding & Executive Director, VART Consulting Pvt, Ltd, Delhi-NCR & Mumbai, India

^7^ Postgraduate Institute of Medical Education and Research (PGIMER), Chandigarh, India ^&^ Corresponding author

E-mail: sonugoel007@yahoo.co.in

# Background

Hypertension is a significant public health concern globally and in India, contributing substantially to the burden of cardiovascular diseases. Early detection through appropriate screening practices is critical to mitigating its long-term impact. However, there is limited evidence on age-specific screening practices for hypertension in the Indian population. This study aims to assess hypertension screening practices across different age groups and identify associated socio-demographic, behavioral, and physiological factors to inform effective public health strategies.

# Methods

A cross-sectional study was conducted using data from the fifth National Family Health Survey (NFHS5), which included 1,01,839 men and 7,24,115 women aged 15 years and above. Hypertension was defined based on systolic blood pressure (SBP) ≥140 mmHg and/or diastolic blood pressure (DBP) ≥90 mmHg. Multivariate logistic regression was used to analyze associations between hypertension and various socio-demographic and behavioral factors. Receiver Operating Characteristic (ROC) analysis determined the optimal age for initiating hypertension screening.

# Results

The prevalence of hypertension increased linearly with age, with a significant rise observed in the 35-39 years age group. Factors significantly associated with hypertension included marital status, religion, education, wealth index, alcohol consumption, and waist-hip ratio. ROC analysis identified 35 years as the optimal age for initiating routine hypertension screening in both men and women. Screening in this age group showed balanced sensitivity and specificity for early detection of hypertension.

# Conclusions

This study highlights the need to revise hypertension screening policies in India, recommending initiation at 35 years to improve early detection and management. Addressing socio-demographic and lifestyle determinants through targeted interventions is critical to achieving national hypertension control goals.

**Keywords:** Hypertension Screening, Lifestyle Factors, Public Health Policy, Non-Communicable Diseases and Cardiovascular Risk

**Introduction:**

Worldwide, the prevalence of hypertension among adults (30-79 years) in 2019 was 32% in women and 34% in men(1). Among those with hypertension, 41% of women and 51% of men did not have a previous diagnosis of hypertension(1). Less than half of the people with hypertension had taken treatment leading to the treatment rate of 47% in women and 38% in men(1). The hypertension control after taking treatment was achieved for 23% women and 18%men with hypertension(1).As per National Noncommunicable Disease Monitoring Survey (NNMS) in India, the prevalence of hypertension among adults aged 18-69 years was 28.5%. Among them, 27% were aware of their hypertensive status, 14.5% were on treatment for hypertension and 12.6% had blood pressure under control(2).

Globally in 2019, high systolic blood pressure (SBP) was the leading risk factor for attributable deaths, accounting for 10.8 million deaths (19.2% of all deaths in 2019)(3). High SBP was attributed to 5.25 million (20.3%) and 5.60 million (18.2%) deaths among females and males respectively in 2019 (3). The leading risk factor in 2019 by percentage of Disability Adjusted Life Years (DALYs) was attributed to high SBP with 9.3 (8.2 to 10.5), 6.0 (4.9 to 7.1), 16.1 (14.2 to 18.0) and 19.5 (16.3 to 22.7) for all ages, 25-49 years, 50-74 years and 75 years and above respectively(3). In most parts of southeast Asia, 10% to 20% of DALYs were attributable to high SBP(3).

International Society of Hypertension (ISH) recommended that for those with normal blood pressure (BP) level (<130/85mmHg), BP would be re-measured after 1 year and 3 years in those with risk factors and without risk factors respectively(4). US Preventive Services Task Force (USPSTF) acknowledged the lack of evidence for optimal screening intervals and recommended the screening practice of once a year among those adults aged 40 and above and also in adults with risk factors and every 3-5 years among adults aged 18-39 years without any risk factors for hypertension(5). While several LMICs face a growing burden of hypertension, few have conducted large-scale, nationally representative studies to determine an age threshold for screening. A study in Nepal by Dhungana et al. (2022) noted that younger adults aged 15–29 years were significantly less likely to have been screened for hypertension, highlighting a missed opportunity for early detection in LMICs(6). However, this study did not provide a precise age cutoff for initiating screening. Our study is among the first in India and similar LMIC settings to use ROC analysis to empirically determine an optimal screening age.

In India, the recommendation under standard treatment guidelines for hypertension was to re-check BP in 2 years among persons with normal BP (<130/80mmHg) and in 1 year for persons with high normal BP(7). Under the National Programme for Prevention and Control of Cancer, DM, Cardiovascular Diseases and Stroke (NPCDCS), the recommended practice was to screen once a yearfor aged 30 years and above (8). Despite India's recommendation of initiating hypertension screening at 30 years under NPCDCS, there is no age-stratified evidence to support this threshold from nationally representative datasets. There is a need for sensitizing the health care providers to screen for high BP at optimum intervals without the need for unnecessary screening and overburdening them(9).

Among hypertensive patients, only 76% had ever received a BP screening according to NFHS-4(10) and 64% had not checked their BP in the last one year in Puducherry, India(11). In Nepal, the prevalence of hypertension screening was 65.9% among the hypertensive individuals(12). Hypertension screening was significantly lower among those in the age group of 15 to 29 years (52.2%), those who were never married (54.4%) and those with no formal schooling (59.6%)(12). In Germany, 37.5% of men and 21.9% of women did not have BP measurement within the last one year among non hypertensive individuals(13). In Canada, 2.6% of the individuals did not have BP measurement ever and 9.0% did not have their BP assessed in the last 2 years(14). In America, 87.4% of individuals of aged 18 to 39 years have never measured their BP at home(13). Even though there is a policy for regular BP measurement with every country(13), there is a gap in the implementation of these policies.

Evidence suggests that SBP increases along with age in a linear relationship whereas DBP increases before 45 years of age and then decreases afterwards in a curvilinear fashion(15). However the relationship between and age varies from one country to another depending on the contributing factors(15). Hence there is a need to identify the relationship between age and BP and to find the appropriate age at which the screening is required for early diagnosis.

The Government of India has adopted a national multi-sectoral action plan for the prevention and control of non-communicable diseases and set a target of 25% relative reduction in the prevalence of raised blood pressure by 2025(16). Since hypertension is an asymptomatic condition in most of the individuals, it can increase the overall risk of cardio-vascular diseases (CVD) and lead to life threatening complications like heart attack, stroke and renal failure(7). Early diagnosis of high BP is achieved by appropriately screening the people at adequate intervals. Screening for high BP at an appropriate interval and at an appropriate age will not only help the individuals to lower the CVD risk factors but also in attaining the Government’starget towards reducing the high BP prevalence in the entire community.

There is a paucity of evidence to suggest the appropriate screening practice for a particular age group from a nation-wide representative sample, although there are some recommendations existing depending on the blood pressure values and for all age groups in general. In this regard, the present study has been planned with the objective of determining the hypertension screening practice across different age groups among the Indian population from a nationwide representative sample (NFHS-5).The objectives of the current study were to assess the hypertension screening practice across different age groups in Indian adults and to identify the factors associated with hypertension among Indian adults

# Materials and Methods

*Study design:*

This cross sectional study utilizes secondary data from the fifth National Family Health Survey (NFHS5), which is the Indian version of the Demographic and Health Survey conducted regularly in more than 90 countries worldwide. NFHS is a collaborative project supported by the International Institute of

Population Sciences (IIPS) in Mumbai, India; International Classification of Functioning, Disability and Health (ICF) in Calverton, Maryland, USA, and East West Center in Honolulu, Hawaii, USA. The Ministry of Health and Family Welfare (MoHFW) of the Government of India has appointed IIPS as the coordinating and technical guidance agency of NFHS. The survey is funded by the United States Agency for International Development (USAID) with additional support from the United Nations Children’s Fund (UNICEF). NFHS uses four different types of questionnaires such as household, man’s, woman’s and biomarker in 19 languages using the Computer Assisted Personal Interviewing (CAPI). The fieldwork for NFHS-5 in India was done in two phases. Phase one took place between June 17, 2019, and January 30, 2020, which covered 17 states and 5 Union Territories. Phase two was conducted between January 2, 2020 and April 30, 2021, which covered 11 states and 3 Union Territories. A total of 17 Field Agencies gathered data from 6,36,699 households, 1,01,839 men and 7,24,115 women.

## Data source

The current data analysis uses the men’s questionnaire and women’s questionnaire from NFHS- 5, mainly focusing on male and female datasets. It focuses on the various independent factors associated with hypertension (dependent variable) among Indian adults.Analyzing the male and female factors separately can provide a clear understanding on the social and cultural factors associated with hypertension. Potential confounding variables such as dietary sodium intake, psychological stress, and family history of hypertension were not included due to limitations in the NFHS-5 data collection instruments. It examines the ideal age points at which the Indian adult population has to be screened for hypertension. Usually, the age cut off for screening hypertension has been taken as 30 years and the evidence provided through a nationwide survey would be more convincing regarding the appropriate age cut off. The total sample size utilized for this secondary data analysis was 1,01,839 men and 7,24,115 women aged 15 years and above. Since there are missing values, each variable in this analysis has a different sample size, as mentioned in the results section.

## Ethical approval

The research was conducted using a pre-existing, anonymised data from a public source, which did not require direct involvement of the patients or the general public. Since the dataset did not contain any personally identifiable information about the study participants, a formal ethics committee approval was not mandatory. However, the ethics committee approval was obtained from the Institutional Ethics Committee of the Post Graduate Institute of Medical Education and Research (PGIMER) in Chandigarh (approval number IEC-08/2022-2535, dated 17.08.2022).

## Definitions

As per the guidelines of World Health Organisation (WHO) and American Heart Association (AHA), the study describes elevated blood pressure as having a systolic blood pressure of 140 mmHg or higher and/or a diastolic blood pressure of 90 mmHg or more(17). The raised risk of metabolic complications was considered when the waist-hip ratio (WHR) was 0.90 or higher for men and 0.85 or higher for women(18). Regions were divided into 6 categories namely north (Chandigarh, Delhi, Haryana, Himachal Pradesh, Jammu & Kashmir, Punjab, Rajasthan, Uttarakhand), central (Chattisgarh, Madhya Pradesh, Uttar Pradesh), east (Bihar, Jharkhand, Odisha and West Bengal), northeast (Arunachal Pradesh, Assam, Manipur, Meghalaya, Mizoram, Nagaland, Sikkim and Tripura), west (Dadra & Nagar Haveli, Daman & Diu, Goa, Gujarat, Maharashtra) and south (Andaman & Nicobar Islands, Andhra Pradesh, Karnataka, Kerala, Lakshadweep, Puducherry, Tamil Nadu and Telangana).

## Statistical analysis

Hypertension was identified as the outcome variable in the study. The average of three blood pressure readings that were collected during the NFHS was considered for the study. The quantitative blood pressure values were then converted into a dichotomous variable as presence or absence of hypertension. Sampling weights were incorporated into the data to address the differential probabilities of selection and participation.

Statistical analysis was performed using IBM SPSS version 20. The data were presented in the form of mean with 95% confidence interval (CI), frequency and percentage. The associations were evaluated using multivariable logistic regression analysis to calculate the adjusted odds ratio and 95% CI of raised blood pressure with age category (8 categories), marital status, religion, educational status, wealth index (5 categories), occupation, residence, region (6 categories), caste, waist-hip ratio, alcohol use, tobacco use, fruit intake, fried food intake and co-morbidities. The fitness of the dataset was assessed using the Hosmer and Lemeshow test and the Goodness of Fit test. Receiver Operating Characteristics (ROC) Curve was used to evaluate and compare the performance of classification models (age) where the outcome variable is binary (raised blood pressure: present/absent). Sensitivity and specificity was used to describe the diagnostic ability of the screening test. The Youden index was used as a summary measure of the ROC curve to select the optimal cut off point for the age within the age group.A p value of less than 0.05 was considered as significant. Missing data were handled by listwise deletion. Participants with missing values for outcome or explanatory variables were excluded from respective analyses. No imputation was performed. Sample sizes for each analysis are reported in the tables to reflect variations due to missing data.

**Results:**

A total of 1,01,839 men and 7,24,115 women participants were included in the NFHS-5 study. Since the missing values were excluded from the study, the sample size varied across different variables. Table 1 and Table 3 highlight the key findings from adult male population. The systolic and diastolic blood pressure increases with increasing age as shown in Figure 1 and Figure 2, which is also evident from the statistically significant trends in the increasing odds ratio. Though SBP does not have any association with marital status, the DBP is showing a significant association with marital status. Married men are having an increased risk of 1.12 times for developing raised DBP compared to never married men. Muslim men are 10% and 14% lesser risk of developing raised SBP and DBP respectively, compared

Hindu men. Christian men are 11% lesser risk of developing raised DBP whereas Others category (Sikh, Buddhist, Jain and Jewish) men are having 1.29 times higher risk of developing raised SBP compared to Hindu men. With an increase in educational status, the odds of developing raised SBP and DBP also increases. When compared to no education men, the primary, secondary and higher educated men show

1.14,1.20 and 1.01 times of having higher SBP. The primary, secondary and higher educated men show 1.09, 1.24 and 1.12 times of having higher DBP in comparison with no education men. There is a linear trend in wealth index for having raised SBP and DBP. Richer and richest quintile shows 1.12 and 1.14 times of higher risk of developing raised SBP compared to poorest quintile. Poorer, middle, richer and richest quintile shows 1.17, 1.32, 1.32 and 1.35 times respectively of developing raised DBP compared to poorest quintile. Though the employed men has 5% and 3% lesser risk of developing raised SBP and DBP, the association was not found to be statistically significant. The rural men have 8% lower risk of developing raised SBP and DBP compared to urban men. Men belonging to east zone have 34% and 33% lesser risk of developing raised SBP and DBP respectively, compared to men from north zone. West zone has 25% lesser risk of raised SBP whereas south zone has 1.27 times higher risk of raised DBP. Men belonging to ST caste have 1.15 times higher risk developing raised SBP whereas OBC and Others has 11% and 9% lesser risk of developing raised DBP respectively.

**Figure 1: Male systolic blood pressure across different age group in the study population**

# Figure 2: Male diastolic blood pressure across different age group in the study population

The men having WHR more than 0.90 have 1.46 and 1.62 times higher risk of developing raised SBP and DBP respectively. Men having a history of alcohol consumption have 1.28 and 1.50 times higher risk of developing raised SBP and DBP respectively. Men having a history of tobacco use 1.20 and 1.05 times higher risk of developing raised SBP and DBP respectively, though the association between raised DBP and tobacco use was not significant. Regarding fruit intake, daily, weekly and occasionally consuming men have 20%. 9% and 15% reduced risk of developing raised DBP respectively, compared to never category men. Regarding fried food intake, daily consuming men have 1.18 times increased risk of developing raised SBP, compared to never category men. Daily, weekly and occasional consumers of fried food have 10%, 9% and 7% reduced risk of developing raised DBP respectively, compared to never category men. There is no association observed between men with co-morbidities such as diabetes and cardio-vascular diseases and raised SBP and DBP.

# Table 1: Distribution of systolic and diastolic blood pressure among males according to sociodemographic, behavioral and physiological characteristics of the study population, NFHS-5, 201921, India (N=1,01,839)

| **Characteristics** | **N** | **(%)** | **Systolic blood pressure** | | **Diastolic blood pressure** | |
| --- | --- | --- | --- | --- | --- | --- |
|  |  |  | **Weighted mean** | **95%CI** | **Weighted mean** | **95%CI** |
| **Socio-demography** |  |  |  |  |  |  |
| **Age category (in years)** |  |  |  |  |  |  |
| 15-19 | 14337 | 14.1 | 115.09 | 114.91-115.28 | 72.86 | 72.72-73.01 |
| 20-24 | 12473 | 12.2 | 119.14 | 118.95-119.34 | 76.53 | 76.37-76.68 |
| 25-29 | 12410 | 12.2 | 120.68 | 120.49-120.88 | 79.04 | 78.89-79.19 |
| 30-34 | 11592 | 11.4 | 122.63 | 122.41-122.84 | 81.00 | 80.84-81.17 |
| 35-39 | 11641 | 11.4 | 123.55 | 123.31-123.78 | 82.25 | 82.08-82.43 |
| 40-44 | 9667 | 9.5 | 124.96 | 124.66-125.25 | 83.52 | 83.31-83.72 |
| 45-49 | 10058 | 9.9 | 126.79 | 126.47-127.10 | 84.10 | 83.89-84.30 |

| 50-54 | 7947 | 7.8 | 128.67 | 128.30-129.03 | 84.57 | 84.34-84.80 |
| --- | --- | --- | --- | --- | --- | --- |
| **Marital status** |  |  |  |  |  |  |
| Never married | 31756 | 31.2 | 118.00 | 117.87-118.13 | 75.75 | 75.65-75.85 |
| Currently married | 57031 | 56.0 | 124.22 | 124.11-124.34 | 82.16 | 82.08-82.24 |
| Widowed /Separated  /Divorced | 1337 | 1.3 | 125.24 | 124.42-126.06 | 82.65 | 82.11-83.19 |
| **Religion** |  |  |  |  |  |  |
| Hindu | 72249 | 70.9 | 122.10 | 122.00-122.20 | 80.03 | 79.95-80.10 |
| Muslim | 13122 | 12.9 | 121.16 | 120.94-121.39 | 78.83 | 78.67-79.00 |
| Christian | 2484 | 2.4 | 123.22 | 122.68-123.76 | 80.87 | 80.48-81.26 |
| Others* | 2269 | 2.2 | 124.09 | 123.48-124.69 | 81.27 | 80.83-81.70 |
| **Educational status** |  |  |  |  |  |  |
| No education | 10893 | 10.7 | 122.95 | 122.68-123.22 | 80.93 | 80.73-81.12 |
| Primary | 10990 | 10.8 | 122.83 | 122.57-123.10 | 80.65 | 80.46-80.84 |
| Secondary | 51460 | 50.5 | 121.58 | 121.46-121.70 | 79.45 | 79.36-79.54 |
| Higher | 16781 | 16.5 | 122.38 | 122.18-122.57 | 80.17 | 80.03-80.32 |
| **Wealth Index of the household** | |  |  |  |  |  |
| Poorest (I quintile) | 15204 | 14.9 | 120.28 | 120.07-120.49 | 78.18 | 78.02-78.33 |
| Poorer (II quintile) | 17872 | 17.5 | 121.21 | 121.00-121.41 | 79.16 | 79.01-79.31 |
| Middle (III quintile) | 19497 | 19.1 | 122.03 | 121.83-122.22 | 80.08 | 79.94-80.23 |
| Richer (IV quintile) | 20190 | 19.8 | 122.76 | 122.57-122.95 | 80.67 | 80.53-80.81 |
| Richest (V quintile) | 17362 | 17.0 | 123.64 | 123.43-123.84 | 81.12 | 80.96-81.27 |
| **Current occupation** |  |  |  |  |  |  |
| Unemployed | 21208 | 20.8 | 118.27 | 118.10-118.45 | 76.18 | 76.05-76.31 |
| Employed | 68917 | 67.7 | 123.20 | 123.10-123.31 | 81.06 | 80.98-81.13 |
| **Residence** |  |  |  |  |  |  |
| Urban | 30569 | 30.0 | 122.84 | 122.68-122.99 | 80.66 | 80.55-80.77 |
| Rural | 59555 | 58.5 | 121.64 | 121.53-121.75 | 79.52 | 79.44-79.61 |
| **Region** |  |  |  |  |  |  |
| North | 7722 | 7.6 | 124.57 | 124.28-124.87 | 80.90 | 80.68-81.11 |
| Central | 10302 | 10.1 | 123.21 | 122.95-123.46 | 80.31 | 80.12-80.51 |
| East | 23013 | 22.6 | 120.36 | 120.19-120.53 | 78.28 | 78.15-78.40 |
| Northeast | 5000 | 4.9 | 122.87 | 122.49-123.25 | 80.16 | 79.88-80.43 |
| West | 21571 | 21.2 | 121.28 | 121.10-121.46 | 79.70 | 79.56-79.83 |
| South | 22516 | 22.1 | 122.93 | 122.74-123.12 | 81.20 | 81.06-81.35 |
| **Caste** |  |  |  |  |  |  |
| SC | 18473 | 18.1 | 122.04 | 121.83-122.23 | 80.05 | 79.90-80.20 |
| ST | 8366 | 8.2 | 122.37 | 122.07-122.66 | 80.01 | 79.79-80.23 |
| OBC | 38188 | 37.5 | 121.78 | 121.64-121.92 | 79.82 | 79.71-79.92 |
| Others | 25096 | 24.6 | 122.35 | 122.18-122.52 | 79.91 | 79.78-80.03 |
| **Behavioral and Physiological** | |  |  |  |  |  |
| **Waist Hip Ratio category** | |  |  |  |  |  |
| >/=0.90 | 44168 | 43.4 | 124.20 | 124.07-124.33 | 82.01 | 81.91-82.11 |
| <0.90 | 40523 | 39.8 | 119.73 | 119.61-119.86 | 77.67 | 77.57-77.76 |
| **History of Alcohol use** |  |  |  |  |  |  |
| No | 69279 | 68.0 | 121.31 | 121.21-121.41 | 79.12 | 79.04-79.19 |
| Yes | 20845 | 20.5 | 124.49 | 124.30-124.69 | 82.54 | 82.39-82.69 |
| **History of tobacco use** |  |  |  |  |  |  |
| No | 78259 | 76.8 | 121.92 | 121.82-122.02 | 79.74 | 79.67-79.81 |
| Yes | 11866 | 11.7 | 122.87 | 122.62-123.12 | 81.01 | 80.82-81.19 |
| **Frequency of fruit intake** |  |  |  |  |  |  |
| Never | 1490 | 1.5 | 121.10 | 120.37-121.83 | 80.12 | 79.58-80.67 |
| Daily | 10590 | 10.4 | 122.72 | 122.46-122.98 | 80.28 | 80.08-80.48 |
| Weekly | 39478 | 38.8 | 122.38 | 122.25-122.52 | 80.21 | 80.11-80.31 |
| Occasionally | 38567 | 37.9 | 121.55 | 121.41-121.68 | 79.49 | 79.39-79.59 |
| **Frequency of fried food intake** | |  |  |  |  |  |
| Never | 6883 | 6.8 | 122.53 | 122.19-122.86 | 80.68 | 80.44-80.93 |
| Daily | 8018 | 7.9 | 122.46 | 122.15-122.76 | 79.72 | 79.50-79.94 |
| Weekly | 32160 | 31.6 | 121.67 | 121.53-121.82 | 79.62 | 79.51-79.73 |
| Occasionally | 43063 | 42.3 | 122.17 | 122.04-122.30 | 80.04 | 79.94-80.14 |
| **Co-morbidities** |  |  |  |  |  |  |
| Hypertension | 3078 | 3.0 | 132.26 | 131.57-132.94 | 87.58 | 87.12-88.04 |
| Diabetes | 2497 | 2.5 | 127.16 | 126.54-127.78 | 84.24 | 83.82-84.66 |
| Cardiovascular disease | 956 | 0.9 | 122.92 | 121.98-123.86 | 81.41 | 80.76-82.05 |

*Includes Sikh, Buddhist, Jain, Jewish and others

Table 2 and Table 4 highlight the key findings from adult female population. The systolic and diastolic blood pressure of women increases with increasing age as shown in Figure 3 and Figure 4, which is also evident from the statistically significant trends in the increasing odds ratio. Though DBP does not have any association with marital status, the SBP is showing a significant association with marital status. Married women are having 21% reduced risk of developing raised SBP compared to never married women. Muslim women are 1.13 and 1.16times increased risk of developing raised SBP and DBP respectively, compared Hindu women. Others category (Sikh, Buddhist, Jain and Jewish) women are having 1.45 and 1.32 times higher risk of developing raised SBP and DBP respectively, compared to Hindu women. With an increase in educational status, the odds of developing raised SBP and DBP decreases. When compared to no education women, the primary, secondary and higher educated women show 9%, 8% and 28% reduced risk having higher SBP. The higher educated women show 20%reduced risk having higher DBP in comparison with no education women. Middle, richer and richest quintile shows 1.13, 1.25 and 1.23 times of higher risk of developing raised DBP compared to poorest quintile. However, there was no association observed between SBP and wealth index among women. The employed women are 14% and 5% lesser risk of developing raised SBP and DBP respectively, compared to unemployed women. There was no association observed between raised BP and residence among women. Women belonging to central, north-east and south zone have 1.29, 1.28 and 1.20 times increasedrisk of developing raised SBP respectively, compared to women from north zone. Similarly, women belonging to central, north-east and south zone have 1.22, 1.17 and 1.32 times increased risk of developing raised DBP respectively. Women belonging to ST caste have 1.27 and 1.16 times higher risk developing raised SBP and DBP respectively, whereas women belonging to OBC have8% lesser risk of developing raised DBP.

**Figure 3: Female systolic blood pressure across different age group in the study population**

# Figure 4: Female diastolic blood pressure across different age group in the study population

The women having WHR more than 0.85 have 1.38 and 1.38 times higher risk of developing raised SBP and DBP respectively. Women having a history of alcohol consumption have 1.44 and 1.56 times higher risk of developing raised SBP and DBP respectively. There was no association observed between raised BP and tobacco use among women. There was no association observed between raised BP and fruit intake among women. Regarding fruit intake, daily, weekly and occasionally consuming men have 20%. 9% and 15% reduced risk of developing raised DBP respectively, compared to never category men. Regarding fried food intake, weekly and occasional consuming women have 24% and 19% reduced risk of developing raised SBP, compared to never category women. Daily, weekly and occasional consumers of fried food have 13%, 17% and 14% reduced risk of developing raised DBP respectively, compared to never category women. Women with diabetes have 1.53 and 1.60 times increased risk of developing raised SBP and DBP respectively.

# Table 2: Distribution of systolic and diastolic blood pressure among females according to sociodemographic, behavioral and physiological characteristics of the study population, NFHS-5, 201921, India (N=7,24,115)

| **Characteristics** | **N** | **(%)** | **Systolic blood pressure** | | **Diastolic blood pressure** | |
| --- | --- | --- | --- | --- | --- | --- |
|  |  |  | **Weighted mean** | **95%CI** | **Weighted mean** | **95%CI** |
| **Socio-demography** |  |  |  |  |  |  |
| **Age category (in years)** |  |  |  |  |  |  |
| 15-19 | 111820 | 15.4 | 109.47 | 109.40-109.53 | 72.34 | 72.29-72.39 |
| 20-24 | 109961 | 15.2 | 110.66 | 110.59-110.72 | 73.99 | 73.94-74.04 |
| 25-29 | 108578 | 15.0 | 112.42 | 112.35-112.49 | 75.84 | 75.79-75.89 |
| 30-34 | 93505 | 12.9 | 115.09 | 115.01-115.17 | 77.93 | 77.87-77.98 |
| 35-39 | 90926 | 12.6 | 117.86 | 117.77-117.95 | 79.68 | 79.62-79.75 |
| 40-44 | 76627 | 10.6 | 121.43 | 121.32-121.53 | 81.25 | 81.18-81.32 |

| 45-49 | 79726 | 11.0 | 124.71 | 124.59-124.83 | 82.27 | 82.20-82.34 |
| --- | --- | --- | --- | --- | --- | --- |
| **Marital status** |  |  |  |  |  |  |
| Never married | 155694 | 21.5 | 110.37 | 110.31-110.42 | 73.46 | 73.42-73.50 |
| Currently married | 486713 | 67.2 | 116.51 | 116.47-116.55 | 78.14 | 78.11-78.16 |
| Widowed /Separated  /Divorced | 28736 | 4.0 | 120.02 | 119.83-120.21 | 80.42 | 80.30-80.54 |
| **Religion** |  |  |  |  |  |  |
| Hindu | 550204 | 76.0 | 115.09 | 115.05-115.13 | 77.08 | 77.06-77.11 |
| Muslim | 86887 | 12.0 | 115.80 | 115.71-115.90 | 77.28 | 77.21-77.34 |
| Christian | 15773 | 2.2 | 114.55 | 114.31-114.79 | 77.26 | 77.09-77.41 |
| Others* | 18280 | 2.5 | 117.60 | 117.39-117.82 | 78.46 | 78.32-78.61 |
| **Educational status** |  |  |  |  |  |  |
| No education | 152941 | 21.1 | 118.82 | 118.74-118.89 | 79.32 | 79.27-79.37 |
| Primary | 79627 | 11.0 | 117.47 | 117.37-117.57 | 78.68 | 78.62-78.75 |
| Secondary | 336361 | 46.5 | 113.84 | 113.79-113.88 | 76.18 | 76.14-76.21 |
| Higher | 102214 | 14.1 | 112.74 | 112.66-112.82 | 75.91 | 75.85-75.96 |
| **Wealth Index of the household** | |  |  |  |  |  |
| Poorest (I quintile) | 125420 | 17.3 | 115.07 | 114.99-115.14 | 76.55 | 76.50-76.61 |
| Poorer (II quintile) | 135405 | 18.7 | 114.83 | 114.76-114.91 | 76.76 | 76.71-76.81 |
| Middle (III quintile) | 139011 | 19.2 | 114.99 | 114.92-115.06 | 77.15 | 77.10-77.20 |
| Richer (IV quintile) | 140220 | 19.4 | 115.30 | 115.22-115.37 | 77.45 | 77.40-77.50 |
| Richest (V quintile) | 131087 | 18.1 | 116.01 | 115.94-116.09 | 77.80 | 77.75-77.85 |
| **Current occupation** |  |  |  |  |  |  |
| Unemployed | 74844 | 10.3 | 114.77 | 114.67-114.87 | 76.82 | 76.75-76.89 |
| Employed | 25627 | 3.5 | 116.20 | 116.02-116.37 | 78.11 | 77.99-78.22 |
| **Residence** |  |  |  |  |  |  |
| Urban | 212706 | 29.4 | 115.44 | 115.38-115.50 | 77.52 | 77.48-77.56 |
| Rural | 458437 | 63.3 | 115.14 | 115.10-115.18 | 76.97 | 76.95-77.00 |
| **Region** |  |  |  |  |  |  |
| North | 95548 | 13.2 | 117.40 | 117.31-117.48 | 78.04 | 77.98-78.09 |
| Central | 168057 | 23.2 | 116.32 | 116.25-116.38 | 77.54 | 77.49-77.58 |
| East | 152178 | 21.0 | 114.33 | 114.26-114.40 | 76.40 | 76.36-76.45 |
| Northeast | 24576 | 3.4 | 116.89 | 116.71-117.07 | 77.62 | 77.50-77.74 |
| West | 93541 | 12.9 | 114.44 | 114.35-114.53 | 76.88 | 76.82-76.94 |
| South | 137243 | 19.0 | 113.65 | 113.58-113.73 | 76.98 | 76.93-77.03 |
| **Caste** |  |  |  |  |  |  |
| SC | 148158 | 20.5 | 114.53 | 114.46-114.60 | 76.87 | 76.82-76.92 |
| ST | 63553 | 8.8 | 116.57 | 116.47-116.68 | 77.57 | 77.50-77.65 |
| OBC | 290700 | 40.1 | 114.94 | 114.89-114.99 | 76.98 | 76.95-77.02 |
| Others | 168734 | 23.3 | 115.86 | 115.80-115.93 | 77.52 | 77.47-77.56 |
| **Behavioral and physiological** | |  |  |  |  |  |
| **Waist Hip Ratio category** | |  |  |  |  |  |
| >/=0.85 | 382488 | 52.8 | 116.87 | 116.82-116.91 | 78.06 | 78.03-78.09 |
| <0.85 | 257040 | 35.5 | 112.80 | 112.75-112.85 | 75.78 | 75.75-75.82 |
| **History of Alcohol use** |  |  |  |  |  |  |
| No | 666052 | 92.0 | 115.20 | 115.17-115.24 | 77.12 | 77.10-77.15 |
| Yes | 5092 | 0.7 | 119.76 | 119.32-120.21 | 80.38 | 80.09-80.68 |
| **History of tobacco use** |  |  |  |  |  |  |
| No | 670500 | 92.6 | 115.24 | 115.20-115.27 | 77.15 | 77.12-77.17 |
| Yes | 644 | 0.1 | 115.62 | 114.43-116.80 | 77.57 | 76.83-78.31 |
| **Frequency of fruit intake** | |  |  |  |  |  |
| Never | 10901 | 1.5 | 115.49 | 115.22-115.77 | 77.44 | 77.25-77.62 |
| Daily | 81322 | 11.2 | 114.68 | 114.59-114.78 | 76.95 | 76.89-77.02 |
| Weekly | 247699 | 34.2 | 115.23 | 115.17-115.28 | 77.20 | 77.16-77.24 |
| Occasionally | 331221 | 45.7 | 115.37 | 115.32-115.42 | 77.15 | 77.12-77.18 |
| **Frequency of fried food intake** | |  |  |  |  |  |
| Never | 29516 | 4.1 | 116.03 | 115.86-116.20 | 77.79 | 77.67-77.90 |
| Daily | 49013 | 6.8 | 115.10 | 114.97-115.23 | 77.05 | 76.96-77.13 |
| Weekly | 237557 | 32.8 | 115.13 | 115.07-115.19 | 77.05 | 77.01-77.09 |
| Occasionally | 355057 | 49.0 | 115.26 | 115.22-115.31 | 77.17 | 77.14-77.21 |
| **Co-morbidities** |  |  |  |  |  |  |
| Hypertension | 32171 | 4.4 | 124.18 | 123.97-124.40 | 82.96 | 82.83-83.10 |
| Diabetes | 12725 | 1.8 | 123.84 | 123.54-124.14 | 82.51 | 82.34-82.69 |
| Cardiovascular disease | 4871 | 0.7 | 117.99 | 117.54-118.43 | 79.24 | 78.95-79.53 |

*Includes Sikh, Buddhist, Jain, Jewish and others

# Table 3: Multivariate logistic regression analysis to determine the factors associated with elevated systolic and diastolic blood pressure among male, NFHS-5, 2019-21, India

|  | **SBP (>/=140mmHg)** | | | **DBP (>/=90mmHg)** | | |
| --- | --- | --- | --- | --- | --- | --- |
| **Characteristics** | **OR** | **95% CI** | | **OR** | **95% CI** | |
|  |  | **Lower** | **Upper** |  | **Lower** | **Upper** |
| **Socio-demographic characteristics** |  |  |  |  |  |  |
| **Age category (in years)** |  |  |  |  |  |  |
| 15-19 | 1 |  |  | 1 |  |  |
| 20-24 | 2.64* | 2.22 | 3.14 | 2.14* | 1.86 | 2.46 |
| 25-29 | 3.13* | 2.62 | 3.75 | 2.98* | 2.58 | 3.43 |
| 30-34 | 4.23* | 3.51 | 5.10 | 4.83* | 4.18 | 5.58 |

| 35-39 | 5.99* | 4.97 | 7.22 | 6.50* | 5.62 | 7.53 |
| --- | --- | --- | --- | --- | --- | --- |
| 40-44 | 9.49* | 7.88 | 11.42 | 8.74* | 7.55 | 10.13 |
| 45-49 | 12.51* | 10.41 | 15.04 | 9.76* | 8.43 | 11.30 |
| 50-54 | 17.29* | 14.36 | 20.81 | 10.56* | 9.10 | 12.25 |
| **Marital status** |  |  |  |  |  |  |
| Never married | 1 |  |  | 1 |  |  |
| Currently married | 0.95 | 0.86 | 1.05 | 1.12* | 1.04 | 1.21 |
| Widowed /Separated /Divorced | 1.09 | 0.90 | 1.32 | 1.15 | 0.98 | 1.36 |
| **Religion** |  |  |  |  |  |  |
| Hindu | 1 |  |  | 1 |  |  |
| Muslim | 0.90* | 0.83 | 0.98 | 0.86* | 0.81 | 0.92 |
| Christian | 0.92 | 0.79 | 1.06 | 0.89* | 0.78 | 1.00 |
| Others* | 1.29* | 1.11 | 1.49 | 1.00 | 0.88 | 1.13 |
| **Educational status** |  |  |  |  |  |  |
| No education | 1 |  |  | 1 |  |  |
| Primary | 1.14* | 1.04 | 1.25 | 1.09* | 1.01 | 1.18 |
| Secondary | 1.20* | 1.11 | 1.30 | 1.24* | 1.16 | 1.33 |
| Higher | 1.01 | 0.91 | 1.12 | 1.12* | 1.03 | 1.21 |
| **Wealth Index of the household** |  |  |  |  |  |  |
| Poorest (I quintile) | 1 |  |  | 1 |  |  |
| Poorer (II quintile) | 1.01 | 0.93 | 1.11 | 1.17* | 1.08 | 1.26 |
| Middle (III quintile) | 1.07 | 0.97 | 1.18 | 1.32* | 1.22 | 1.43 |
| Richer (IV quintile) | 1.12* | 1.01 | 1.23 | 1.32* | 1.22 | 1.43 |
| Richest (V quintile) | 1.14* | 1.02 | 1.28 | 1.35* | 1.23 | 1.49 |
| **Current occupation** |  |  |  |  |  |  |
| Unemployed | 1 |  |  | 1 |  |  |
| Employed | 0.95 | 0.88 | 1.03 | 0.97 | 0.91 | 1.04 |
| **Residence** |  |  |  |  |  |  |
| Urban | 1 |  |  | 1 |  |  |
| Rural | 0.92* | 0.86 | 0.97 | 0.92* | 0.88 | 0.97 |
| **Region** |  |  |  |  |  |  |
| North | 1 |  |  | 1 |  |  |
| Central | 1.05 | 0.93 | 1.17 | 1.06 | 0.97 | 1.17 |
| East | 0.66* | 0.59 | 0.73 | 0.67* | 0.62 | 0.74 |
| Northeast | 1.01 | 0.88 | 1.16 | 0.97 | 0.86 | 1.09 |
| West | 0.75* | 0.68 | 0.83 | 0.95 | 0.88 | 1.04 |
| South | 1.03 | 0.93 | 1.13 | 1.27* | 1.17 | 1.38 |
| **Caste** |  |  |  |  |  |  |
| SC | 1 |  |  | 1 |  |  |
| ST | 1.15* | 1.04 | 1.28 | 1.04 | 0.95 | 1.12 |
| OBC | 0.95 | 0.89 | 1.02 | 0.89* | 0.84 | 0.94 |
| Others | 1.01 | 0.93 | 1.09 | 0.91* | 0.85 | 0.97 |
| **Behavioral and physiological characteristics** |  |  |  |  |  |  |
| **Waist Hip Ratio (WHR) category** |  |  |  |  |  |  |
| <0.90 | 1 |  |  | 1 |  |  |
| >/=0.90 | 1.46* | 1.39 | 1.54 | 1.62* | 1.55 | 1.69 |
| **History of Alcohol use** |  |  |  |  |  |  |
| No | 1 |  |  | 1 |  |  |
| Yes | 1.28* | 1.20 | 1.35 | 1.50* | 1.43 | 1.57 |
| **History of tobacco use** |  |  |  |  |  |  |
| No | 1 |  |  | 1 |  |  |
| Yes | 1.20* | 1.11 | 1.29 | 1.05 | 0.98 | 1.11 |
| **Frequency of fruit intake** |  |  |  |  |  |  |
| Never | 1 |  |  | 1 |  |  |
| Daily | 1.15 | 0.93 | 1.42 | 0.80* | 0.68 | 0.95 |
| Weekly | 1.09 | 0.89 | 1.33 | 0.91 | 0.78 | 1.06 |
| Occasionally | 1.05 | 0.86 | 1.29 | 0.85* | 0.73 | 0.99 |
| **Frequency of fried food intake** |  |  |  |  |  |  |
| Never | 1 |  |  | 1 |  |  |
| Daily | 1.18* | 1.04 | 1.33 | 0.90* | 0.81 | 0.99 |
| Weekly | 0.99 | 0.89 | 1.09 | 0.91* | 0.84 | 0.98 |
| Occasionally | 1.08 | 0.99 | 1.19 | 0.93* | 0.86 | 1.00 |
| **Co-morbidities** |  |  |  |  |  |  |
| Diabetes | 1.12 | 1.00 | 1.25 | 1.01 | 0.92 | 1.12 |
| Cardiovascular disease | 0.81 | 0.65 | 1.00 | 0.84 | 0.70 | 1.01 |

*p value<0.05

# Table 4: Multivariate logistic regression analysis to determine the factors associated with elevated systolic and diastolic blood pressure among female, NFHS-5, 2019-21, India

|  | **SBP (>/=140mmHg)** | | | **DBP (>/=90mmHg)** | | |
| --- | --- | --- | --- | --- | --- | --- |
| **Characteristics** | **OR** | **95% CI** | | **OR** | **95% CI** | |
|  |  | **Lower** | **Upper** |  | **Lower** | **Upper** |
| **Socio-demographic characteristics** |  |  |  |  |  |  |
| **Age category (in years)** |  |  |  |  |  |  |
| 15-19 | 1 |  |  | 1 |  |  |

| 20-24 | 1.88* | 1.40 | 2.52 | 1.71* | 1.45 | 2.01 |
| --- | --- | --- | --- | --- | --- | --- |
| 25-29 | 3.89* | 2.91 | 5.21 | 2.49* | 2.10 | 2.95 |
| 30-34 | 7.25* | 5.43 | 9.68 | 4.10* | 3.46 | 4.86 |
| 35-39 | 12.76* | 9.60 | 16.96 | 6.77* | 5.73 | 8.00 |
| 40-44 | 24.53* | 18.48 | 32.55 | 9.01* | 7.61 | 10.66 |
| 45-49 | 37.07* | 27.97 | 49.13 | 11.16* | 9.44 | 13.20 |
| **Marital status** |  |  |  |  |  |  |
| Never married | 1 |  |  | 1 |  |  |
| Currently married | 0.79* | 0.66 | 0.95 | 1.05 | 0.93 | 1.19 |
| Widowed /Separated /Divorced | 0.95 | 0.77 | 1.18 | 1.12 | 0.96 | 1.30 |
| **Religion** |  |  |  |  |  |  |
| Hindu | 1 |  |  | 1 |  |  |
| Muslim | 1.13* | 1.03 | 1.25 | 1.16* | 1.08 | 1.25 |
| Christian | 0.90 | 0.74 | 1.11 | 0.97 | 0.84 | 1.13 |
| Others | 1.45* | 1.22 | 1.71 | 1.32* | 1.16 | 1.51 |
| **Educational status** |  |  |  |  |  |  |
| No education | 1 |  |  | 1 |  |  |
| Primary | 0.91 | 0.83 | 1.01 | 1.07 | 0.99 | 1.15 |
| Secondary | 0.92* | 0.84 | 1.00 | 0.99 | 0.93 | 1.06 |
| Higher | 0.72* | 0.62 | 0.82 | .80* | 0.72 | 0.88 |
| **Wealth Index of the household** |  |  |  |  |  |  |
| Poorest (I quintile) | 1 |  |  | 1 |  |  |
| Poorer (II quintile) | 0.96 | 0.86 | 1.06 | 1.08 | 0.99 | 1.17 |
| Middle (III quintile) | 1.07 | 0.96 | 1.20 | 1.13* | 1.04 | 1.23 |
| Richer (IV quintile) | 1.13 | 1.00 | 1.27 | 1.25* | 1.14 | 1.37 |
| Richest (V quintile) | 1.12 | 0.98 | 1.29 | 1.23* | 1.11 | 1.37 |
| **Current occupation** |  |  |  |  |  |  |
| Unemployed | 1 |  |  | 1 |  |  |
| Employed | 0.86* | 0.80 | 0.92 | 0.95 | 0.90 | 1.00 |
| **Residence** |  |  |  |  |  |  |
| Urban | 1 |  |  | 1 |  |  |
| Rural | 0.98 | 0.90 | 1.06 | 0.95 | 0.90 | 1.01 |
| **Region** |  |  |  |  |  |  |
| North | 1 |  |  | 1 |  |  |
| Central | 1.29* | 1.15 | 1.44 | 1.22* | 1.12 | 1.33 |
| East | 1.01 | 0.90 | 1.14 | 0.97 | 0.88 | 1.06 |
| Northeast | 1.28* | 1.07 | 1.54 | 1.17* | 1.01 | 1.35 |
| West | .94 | 0.83 | 1.07 | 1.07 | 0.98 | 1.17 |
| South | 1.20* | 1.07 | 1.34 | 1.32* | 1.21 | 1.43 |
| **Caste** |  |  |  |  |  |  |
| SC | 1 |  |  | 1 |  |  |
| ST | 1.27* | 1.12 | 1.44 | 1.16* | 1.06 | 1.28 |
| OBC | 0.96 | 0.88 | 1.04 | 0.92* | 0.86 | 0.98 |
| Others | 1.10 | 1.00 | 1.22 | 1.03 | 0.95 | 1.10 |
| **Behavioral and physiological characteristics** | |  |  |  |  |  |
| **Waist Hip Ratio (WHR) category** |  |  |  |  |  |  |
| <0.85 | 1 |  |  | 1 |  |  |
| >/=0.85 | 1.38* | 1.28 | 1.48 | 1.38* | 1.31 | 1.46 |
| **History of Alcohol use** |  |  |  |  |  |  |
| No | 1 |  |  | 1 |  |  |
| Yes | 1.44* | 1.13 | 1.84 | 1.56* | 1.28 | 1.89 |
| **History of tobacco use** |  |  |  |  |  |  |
| No | 1 |  |  | 1 |  |  |
| Yes | 1.01 | 0.38 | 2.73 | 0.44 | 0.15 | 1.27 |
| **Frequency of fruit intake** |  |  |  |  |  |  |
| Never | 1 |  |  | 1 |  |  |
| Daily | 0.96 | 0.75 | 1.23 | 0.83 | 0.69 | 1.00 |
| Weekly | 1.00 | 0.79 | 1.26 | 0.88 | 0.74 | 1.05 |
| Occasionally | 0.93 | 0.74 | 1.18 | 0.85 | 0.72 | 1.02 |
| **Frequency of fried food intake** |  |  |  |  |  |  |
| Never | 1 |  |  | 1 |  |  |
| Daily | 0.85 | 0.71 | 1.01 | 0.87* | 0.75 | 0.99 |
| Weekly | 0.76* | 0.66 | 0.88 | 0.83* | 0.75 | 0.93 |
| Occasionally | 0.81* | 0.71 | 0.93 | 0.86* | 0.77 | 0.96 |
| **Co-morbidities** |  |  |  |  |  |  |
| Diabetes | 1.53* | 1.32 | 1.77 | 1.60* | 1.42 | 1.81 |
| Cardiovascular disease | 1.06 | 0.80 | 1.41 | 1.00 | 0.79 | 1.25 |

*p value<0.05

Table 5, Figure 5 and Figure 6 highlights the cut off age in years for adult men using Receiver Operating Characteristics (ROC) Curve. The ROC has been used to evaluate and compare the performance of age as the screening tool to identify the binary outcome variable (raised blood pressure). The Youden index was used as a summary measure of the ROC curve to select the optimal cut off point for the age within the age group. The ROC analysis yielded an AUC of 0.71 (95% CI: 0.70–0.72) for males and 0.68 (95% CI: 0.67–0.69) for females when using age to predict elevated systolic blood pressure (SBP). The 35 to 39 years age group has shown the least difference between sensitivity and specificity and hence it can be suggested as the ideal screening age among adult men for raised SBP and DBP. Table 6, Figure 7 and Figure 8 highlights the cut off age in years for adult women using ROC Curve. Similar to adult men, the 35 to 39 years age group has shown the least difference between sensitivity and specificity and hence it can be suggested as the ideal screening age among adult women for raised SBP and DBP. The Youden index identified 36.5 years as the age cutoff with the best balance of sensitivity (57.9%) and specificity (49.7%) for men, and similar findings for women, supporting the recommendation of 35 years as a screening initiation point

**Figure 5: Male systolic blood pressure across different age group in the study population**

**Figure 6: Male diastolic blood pressure across different age group in the study population**

**Figure 7: Female systolic blood pressure across different age group in the study population**

**Figure 8: Female diastolic blood pressure across different age group in the study population**

# Table 5:Cut-off in years for males based on ROC curves

| **Age category (in years)** | **SBP (>/=140mmHg)** | | | **DBP (>/=90mmHg)** | | |
| --- | --- | --- | --- | --- | --- | --- |
|  | Cut off | Sensitivity | Specificity | Cut off | Sensitivity | Specificity |
| 15-19 | 16.5 | 70.0% | 41.8% | 17.5 | 52.6% | 61.4% |
| 20-24 | 21.5 | 63.2% | 41.1% | 21.5 | 66.5% | 41.3% |
| 25-29 | 26.5 | 60.1% | 43.2% | 26.5 | 61.2% | 43.5% |
| 30-34 | 31.5 | 60.6% | 44.4% | 31.5 | 57.9% | 44.4% |
| 35-39 | 36.5 | 57.9% | 49.7% | 36.5 | 56.5% | 50.2% |
| 40-44 | 41.5 | 57.0% | 48.2% | 40.5 | 70.3% | 33.3% |
| 45-49 | 46.5 | 57.1% | 49.5% | 46.5 | 55.0% | 49.4% |
| 50-54 | 51.5 | 58.1% | 44.5% | 50.5 | 74.6% | 27.1% |
| 15-54 (overall) | 35.5 | 68.0% | 63.9% | 30.5 | 78.8% | 52.3% |

# Table 6:Cut-off in years for females based on ROC curves

| **Age category (in years)** | **SBP (>/=140mmHg)** | | | **DBP (>/=90mmHg)** | | |
| --- | --- | --- | --- | --- | --- | --- |
|  | Cut off | Sensitivity | Specificity | Cut off | Sensitivity | Specificity |
| 15-19 | 16.5 | 66.3% | 40.4% | 16.5 | 64.2% | 40.4% |
| 20-24 | 21.5 | 64.1% | 40.4% | 21.5 | 65.4% | 40.6% |
| 25-29 | 26.5 | 63.1% | 43.7% | 26.5 | 62.6% | 43.9% |
| 30-34 | 31.5 | 63.1% | 44.5% | 31.5 | 61.1% | 44.7% |
| 35-39 | 36.5 | 60.6% | 47.4% | 36.5 | 57.8% | 47.6% |
| 40-44 | 41.5 | 59.4% | 46.2% | 41.5 | 57.5% | 46.2% |
| 45-49 | 46.5 | 59.3% | 46.4% | 46.5 | 56.9% | 46.1% |
| 15-49 (overall) | 34.5 | 78.2% | 65.4% | 32.5 | 72.5% | 61.5% |

Table 7 presents the diagnostic performance of age as a predictor of hypertension using ROC analysis for both systolic and diastolic blood pressure in males and females. The analysis identifies 36.5 years as the optimal cut-off age for initiating hypertension screening in both sexes, based on the Youden Index.

Among males, the area under the curve (AUC) was 0.71 for systolic BP and 0.69 for diastolic BP, indicating fair discriminatory ability. In females, the AUC values were slightly lower at 0.68 for systolic BP and 0.66 for diastolic BP. Sensitivity and specificity values at this cut-off were reasonably balanced, ranging from 56% to 61% sensitivity and 47% to 50% specificity. These findings support 35–36 years as a data-driven and practical threshold for routine hypertension screening among Indian adults.

Table 7: ROC-Based Diagnostic Performance of Age for Predicting Hypertension in Indian Adults (NFHS-5, 2019–21)

| **Sex** | **Outcome** | **Cut-off Age (years)** | **AUC (95%**  **CI)** | **Sensitivity (%)** | **Specificity (%)** | **Youden Index** |
| --- | --- | --- | --- | --- | --- | --- |
| **Male** | SBP ≥140 mmHg | 36.5 | 0.71 (0.70–  0.72) | 57.9 | 49.7 | 0.076 |
|  | DBP ≥90 mmHg | 36.5 | 0.69 (0.68–  0.70) | 56.5 | 50.2 | 0.067 |
| **Female** | SBP ≥140 mmHg | 36.5 | 0.68 (0.67–  0.69) | 60.6 | 47.4 | 0.08 |
|  | DBP ≥90 mmHg | 36.5 | 0.66 (0.65–  0.67) | 57.8 | 47.6 | 0.054 |

**Discussion:**

The present study utilized data from the NFHS-5 survey, which includes data from 1,01,839 men and 7,24,115 women participants. Hypertension, identified in 28.5% of Indian adults aged 18-69, remains a major public health challenge, contributing significantly to the rising burden of noncommunicable diseases (NCDs) in India(19,20). The findings emphasize the need for targeted screening interventions, particularly focusing on desired age for screening, along with sociodemographic, behavioral, and physiological factors.

Comparison with Global and National Trends

Globally, the prevalence of hypertension among adults was 32% in women and 34% in men in 2019, with 41% of women and 51% of men being unaware of their hypertensive status (19). This study's findings align with global trends, showing a linear increase in blood pressure with age, emphasizing the need for timely detection. The study identifies the age group of 35-39 years as a critical point for initiating hypertension screening, suggesting that approximately 60% of adults in this age group have elevated blood pressure, a statistic that parallels the 60% sensitivity identified in the current study(21).

The World Health Organization (WHO) emphasizes opportunistic screening for all adults in primary care settings but does not recommend a specific age threshold(22). The US Preventive Services Task Force (USPSTF) recommends annual hypertension screening for adults aged ≥40 years or younger adults with risk factors, and every 3–5 years for those aged 18–39 years without risk factors(21). The Indian NPCDCS program currently recommends annual screening starting at 30 years of age(23), but this recommendation lacks age-stratified evidence from large-scale national datasets. Our study contributes by empirically identifying 36.5 years as the optimal cutoff based on ROC analysis, offering the first age-specific evidence from a nationally representative Indian sample (NFHS-5). Compared to prior Indian studies such as Prenissl et al. (2019), which highlighted awareness gaps but did not define an ideal screening age(24), our findings offer direct policy guidance.

In India, the National Family Health Survey (NFHS-5) data highlights a gap in hypertension awareness, with only 27% of hypertensive individuals aware of their condition, 14.5% receiving treatment, and just 12.6% having controlled blood pressure(20). These figures underscore the need for improved screening strategies. This study further suggests that starting screening at 35 years could potentially capture a significant portion of undiagnosed cases, contributing to the national goal of reducing hypertension prevalence by 25% by 2025 (25).This gap in awareness and management is concerning, especially considering the high prevalence of hypertension, which is a major risk factor for cardiovascular diseases, including stroke and heart attacks(26).

One of the critical challenges in managing hypertension in India is the low rate of awareness. Studies have shown that lack of awareness is often due to the asymptomatic nature of hypertension, where individuals do not experience symptoms until significant damage has occurred to vital organs (27). This underlines the importance of regular screening to identify individuals with elevated blood pressure before complications arise.

Moreover, the treatment rates for hypertension are alarmingly low. Even among those diagnosed, the rate of treatment is just 14.5%, indicating significant barriers to accessing healthcare, such as economic constraints, lack of healthcare infrastructure, and inadequate follow-up (28). Cultural beliefs and practices also play a role in influencing health-seeking behavior, with many individuals relying on traditional medicine or home remedies instead of seeking formal medical treatment(29).

The control rate, with only 12.6% of hypertensive individuals maintaining their blood pressure within recommended levels, highlights the challenges in ongoing management and adherence to treatment regimens(20). Poor adherence to prescribed medication, often due to side effects, lack of understanding of the chronic nature of hypertension, and the financial burden of long-term treatment, are significant factors contributing to this issue(30).

Given these challenges, the study suggests that initiating hypertension screening at age 35 could play a crucial role in capturing a significant portion of undiagnosed cases. The age group of 3539 years is identified as a critical period where the prevalence of hypertension begins to rise sharply. Within this age group, the study found that 56.4% of males and 60.9% of females have elevated blood pressure, underscoring the importance of early detection(31).Early screening is essential because evidence shows that hypertension detected and managed early significantly reduces the risk of cardiovascular events later in life. For example, the Framingham Heart Study demonstrated that individuals who developed hypertension by their mid-30s had a markedly higher lifetime risk of cardiovascular disease, including heart failure, myocardial infarction, and stroke(32).

The benefits of early screening are further supported by findings from the SPRINT trial, which showed that intensive blood pressure management in individuals at risk significantly reduced the rates of cardiovascular events and all-cause mortality(33). Additionally, a systematic review of hypertension management strategies in low- and middle-income countries highlighted that early detection through community-based screening programs effectively reduces the burden of hypertension-related complications(34).

Socio-Demographic Determinants

This study reveals a significant association between marital status and diastolic blood pressure (DBP), with married men exhibiting a 12% increased risk of elevated DBP compared to their unmarried counterparts. This finding resonates with previous studies indicating that married individuals, particularly men, may experience higher blood pressure due to marital stress(35). However, this stress-related increase in DBP needs to be balanced against the generally recognized health benefits of marriage, such as better social support and health behaviors(36).

The study also highlights a 10% lower risk of hypertension among Muslims compared to Hindus. This difference could be attributed to religious practices such as fasting during Ramadan, which has been shown to have beneficial effects on cardiovascular health(31). Further, dietary habits prevalent in Muslim communities, such as lower consumption of alcohol and possibly higher consumption of whole grains and legumes, may contribute to this reduced risk (37).

Education and wealth index were also significant predictors of hypertension. The study found that individuals with no formal education had a 20% higher risk of hypertension compared to those with higher education. This is consistent with global data suggesting that lower educational attainment is associated with poorer health outcomes due to factors like reduced health literacy and access to healthcare services (38). Moreover, the wealth index showed a clear gradient, with individuals in the highest quintile being 35% more likely to have elevated DBP, possibly due to lifestyle factors such as higher dietary intake of fats and sugars(39).

Behavioral and Physiological Risk Factors

Behavioral risk factors, particularly alcohol consumption and obesity, showed strong associations with hypertension in this study. Men who consumed alcohol were 28% more likely to have elevated systolic blood pressure (SBP), while those with a high waist-hip ratio (WHR) had a 46% higher risk of elevated SBP and a 62% higher risk of elevated DBP (40). These findings are supported by global statistics that link excessive alcohol consumption to an increased risk of hypertension due to its effects on the cardiovascular system (41).

Obesity, as indicated by WHR, is a well-established risk factor for hypertension, with studies showing that a 5 kg/m² increase in body mass index (BMI) can increase the risk of hypertension by 49% (42). The study's findings that higher WHR correlates with a substantial increase in hypertension risk emphasize the need for weight management interventions as part of hypertension prevention strategies.

Interestingly, the study reports a protective effect of fried food consumption on DBP, particularly in women, with those consuming fried foods weekly having a 24% reduced risk of elevated SBP and a 17% reduced risk of elevated DBP. This finding is contrary to the general understanding that fried foods, typically high in trans fats and sodium, contribute to hypertension(43). This unexpected result suggests that other factors, such as the type of oils used or the presence of other healthy behaviors, might be influencing these outcomes. For instance, some traditional Indian cooking practices involve the use of oils rich in unsaturated fats, which might offset the negative effects of frying(44). Further research is needed to disentangle these associations and understand the complex dietary patterns at play.

The study also highlights a gender difference in the impact of fruit intake on blood pressure, with daily fruit consumption associated with a 20% reduced risk of elevated DBP in men, but not in women. This difference could be due to variations in the types of fruits consumed or the overall dietary context in which fruit is eaten. Fruits are rich in potassium, which has been shown to counteract the effects of sodium and lower blood pressure (45). However, gender differences in nutrient metabolism or hormone levels may modify this protective effect, warranting further investigation.

Implications for Public Health and Policy

In India, the Ministry of Health and Family Welfare (MoHFW) recommends hypertension screening starting at age 30 (25). However, this study suggests that screening should be intensified from age 35 to capture those at risk earlier, as evidence suggests that undiagnosed hypertension in this group leads to increased cardiovascular morbidity later in life (35). Studies have shown that hypertension in individuals aged 35-44 is associated with a significantly higher risk of cardiovascular events, including heart attacks and strokes, compared to those diagnosed later in life(31,36). Furthermore, data from the Framingham Heart Study indicate that individuals who develop hypertension by their mid-30s have a higher lifetime risk of cardiovascular disease, emphasizing the importance of early detection and management(37). Additional research from the INTERHEART study confirms that the early onset of hypertension, particularly before age 40, substantially increases the risk of myocardial infarction, making early and targeted screening interventions crucial (38).

Based on the identified ROC thresholds and age-specific risk profiles, we recommend revising

India’s hypertension screening guidelines to initiate routine screening from 35 years onward for both men and women. This adjustment balances feasibility with early detection impact, especially considering that SBP begins to rise substantially from this age group onward.

The study's findings also highlight the need for integrated public health strategies that address the underlying socio-economic and behavioral determinants of hypertension. For instance, public health campaigns should promote lifestyle changes such as reducing alcohol intake and managing body weight, particularly among younger adults. Given that only 14.5% of hypertensive individuals in India are receiving treatment, there is a clear need for improving access to care and ensuring that screening programs are effectively linked to treatment and follow-up services(20). Moreover, educational interventions aimed at improving health literacy could play a crucial role in reducing hypertension prevalence. The study's findings that lower educational attainment and wealth are associated with higher hypertension risk underscore the importance of addressing these social determinants of health through targeted public health interventions (46).

Limitations and Future Research

While this study provides valuable insights, it has limitations that should be acknowledged. The cross-sectional design limits the ability to establish causality between the identified risk factors and hypertension. Future research should focus on longitudinal studies to better understand the temporal relationships and causality (47). Additionally, the reliance on self-reported data for alcohol and tobacco use may introduce recall bias, potentially leading to underestimation of these behaviors' impact on hypertension (48). We acknowledge the possibility of regional disparities in healthcare access, awareness, and hypertension screening practices across different states and zones in India, which may influence screening rates and outcomes. This warrants further subnational analysis.

The study also did not include detailed dietary assessments, particularly sodium intake, which is a key contributor to hypertension. The average sodium intake in India is estimated to be over 10 grams per day, which is more than double the World Health Organization's recommended limit of 5 grams per day(49). Future studies should incorporate detailed dietary data, including sodium and potassium intake, to better understand their roles in hypertension in the Indian context (50).

**Conclusion:**

This study, based on nationally representative NFHS-5 data, provides robust evidence supporting the initiation of routine hypertension screening at 35 years of age for both men and women in India. Using ROC analysis, we identified 36.5 years as the optimal age cutoff with balanced sensitivity and specificity, suggesting a practical and data-driven revision to current national screening guidelines. While the cross-sectional nature of the dataset limits causal inferences, our findings warrant validation through longitudinal studies to confirm the predictive value of this threshold over time. To improve screening uptake among younger adults—especially in underserved regions—targeted community awareness campaigns, workplace-based screening programs, and mobile health interventions integrated with frontline health worker outreach (e.g., ASHAs) should be prioritized. These strategies can facilitate earlier diagnosis, improve blood pressure control, and reduce the burden of cardiovascular disease across the adult population.

Acknowledgement: None

Conflict of interest: None

**Reference:**

1. Zhou B, Carrillo-Larco RM, Danaei G, Riley LM, Paciorek CJ, Stevens GA, et al. Worldwide trends in hypertension prevalence and progress in treatment and control from 1990 to 2019: a pooled analysis of 1201 population-representative studies with 104 million participants. The Lancet. 2021 Sep 11;398(10304):957–80.
2. Amarchand R, Kulothungan V, Krishnan A, Mathur P. Hypertension treatment cascade in India: results from National Noncommunicable Disease Monitoring Survey. J Hum Hypertens. 2022 May 5;1–11.
3. Murray CJL, Aravkin AY, Zheng P, Abbafati C, Abbas KM, Abbasi-Kangevari M, et al. Global burden of 87 risk factors in 204 countries and territories, 1990–2019: a systematic analysis for the Global Burden of Disease Study 2019. The Lancet. 2020 Oct 17;396(10258):1223–49.
4. Unger T, Borghi C, Charchar F, Khan NA, Poulter NR, Prabhakaran D, et al. 2020 International Society of Hypertension Global Hypertension Practice Guidelines. Hypertension. 2020 Jun;75(6):1334–57.
5. US Preventive Services Task Force. Screening for Hypertension in Adults: US Preventive Services Task Force Reaffirmation Recommendation Statement. JAMA. 2021 Apr 27;325(16):1650–6.
6. Dhungana RR, Pedisic Z, Dhimal M, Bista B, de Courten M. Hypertension screening, awareness, treatment, and control: a study of their prevalence and associated factors in a nationally representative sample from Nepal. Glob Health Action. 2022 Dec 31;15(1):2000092.
7. Hypertension_full.pdf [Internet]. [cited 2022 Aug 4]. Available from: https://nhm.gov.in/images/pdf/guidelines/nrhm-guidelines/stg/Hypertension_full.pdf
8. Module for Multi-Purpose Workers - Prevention, Screening and Control of Common NCDS_2.pdf [Internet]. [cited 2022 Aug 5]. Available from:

https://main.mohfw.gov.in/sites/default/files/Module%20for%20Multi-Purpose%20Workers%20-

%20Prevention%2C%20Screening%20and%20Control%20of%20Common%20NCDS_2.pdf

9. Raghuveer P, Anand T, Tripathy JP, Nirgude AS, Reddy MM, Nandy S, et al. Opportunistic screening for diabetes mellitus and hypertension in primary care settings in Karnataka, India: a few steps forward but still some way to go. F1000Research. 2020 May 6;9:335.
10. Prenissl J, Manne-Goehler J, Jaacks LM, Prabhakaran D, Awasthi A, Bischops AC, et al. Hypertension screening, awareness, treatment, and control in India: A nationally representative crosssectional study among individuals aged 15 to 49 years. PLOS Med. 2019 May 3;16(5):e1002801.
11. Babu V, Sahu SK, Kanungo S. Hypertension control status and quality of care for hypertension among patients availing treatment from private sector: A cross-sectional study in urban field practice area of JIPMER, Puducherry. J Fam Med Prim Care. 2019 Jan;8(1):72–6.
12. Dhungana RR, Pedisic Z, Dhimal M, Bista B, de Courten M. Hypertension screening, awareness, treatment, and control: a study of their prevalence and associated factors in a nationally representative sample from Nepal. Glob Health Action. 2022 Dec 31;15(1):2000092.
13. Diederichs C, Neuhauser H. The frequency and determinants of blood pressure measurement by a health professional in Germany: A cross-sectional study. Medicine (Baltimore). 2019 Apr;98(16):e15093.
14. Amankwah E, Campbell NRC, Maxwell C, Onysko J, Quan H. Why Some Adult Canadians Do Not Have Blood Pressure Measured. J Clin Hypertens. 2007 Dec 19;9(12):944–51.
15. Lin JD, Chen YL, Wu CZ, Hsieh CH, Pei D, Liang YJ, et al. Identification of Normal Blood Pressure in Different Age Group. Medicine (Baltimore). 2016 Apr 8;95(14):e3188.
16. National Multisectoral Action Plan (NMAP) for Prevention and Control of Common NCDs (2017-22)_1.pdf [Internet]. [cited 2022 Aug 5]. Available from:

https://main.mohfw.gov.in/sites/default/files/National%20Multisectoral%20Action%20Plan%20%28N

MAP%29%20for%20Prevention%20and%20Control%20of%20Common%20NCDs%20%28201722%29_1.pdf

17. Hypertension [Internet]. [cited 2024 Aug 7]. Available from: https://www.who.int/newsroom/fact-sheets/detail/hypertension
18. World Health Organization. Waist circumference and waist-hip ratio : report of a WHO expert consultation, Geneva, 8-11 December 2008. 2011 [cited 2024 Aug 7]; Available from: https://iris.who.int/handle/10665/44583
19. Zhou B, Carrillo-Larco RM, Danaei G, Riley LM, Paciorek CJ, Stevens GA, et al. Worldwide trends in hypertension prevalence and progress in treatment and control from 1990 to 2019: a pooled analysis of 1201 population-representative studies with 104 million participants. The Lancet. 2021 Sep 11;398(10304):957–80.
20. Amarchand R, Kulothungan V, Krishnan A, Mathur P. Hypertension treatment cascade in India: results from National Noncommunicable Disease Monitoring Survey. J Hum Hypertens. 2022 May 5;1–11.
21. US Preventive Services Task Force. Screening for Hypertension in Adults: US Preventive Services Task Force Reaffirmation Recommendation Statement. JAMA. 2021 Apr 27;325(16):1650–6.
22. HEARTS: Technical package for cardiovascular disease management in primary health care:

Risk-based CVD management [Internet]. [cited 2025 May 16]. Available from: https://www.who.int/publications/i/item/9789240001367

23. National Programme for Prevention and Control of Non Communicable Diseases (NP-NCD) Guidelines | Ministry of Health and Family Welfare | GOI [Internet]. [cited 2025 May 16]. Available from: https://mohfw.gov.in/?q=Major-Programmes/non-communicable-diseases-injury-trauma/Non-

Communicable-Disease-II/National-Programme-for-Prevention-and-Control-of-Cancer-DiabetesCardiovascular-diseases-and-Stroke-NPCDCS

24. Prenissl J, Manne-Goehler J, Jaacks LM, Prabhakaran D, Awasthi A, Bischops AC, et al. Hypertension screening, awareness, treatment, and control in India: A nationally representative crosssectional study among individuals aged 15 to 49 years. PLoS Med. 2019 May;16(5):e1002801.
25. National Multisectoral Action Plan (NMAP) for Prevention and Control of Common NCDs (2017-22)_1.pdf [Internet]. [cited 2024 Sep 3]. Available from:

https://mohfw.gov.in/sites/default/files/National%20Multisectoral%20Action%20Plan%20%28NMAP

%29%20for%20Prevention%20and%20Control%20of%20Common%20NCDs%20%28201722%29_1.pdf

26. Koeppel L, Dittrich S, Miguel SB, Carmona S, Ongarello S, Vetter B, et al. Addressing the diagnostic gap in hypertension through possible interventions and scale-up: A microsimulation study.

PLOS Med [Internet]. 2022 Dec [cited 2024 Sep 4];19(12). Available from:

https://www.ncbi.nlm.nih.gov/pmc/articles/PMC9725126/

27. Awareness, Treatment, and Control of Hypertension among the Adult Population in Burkina Faso: Evidence from a Nationwide Population-Based Survey - PMC [Internet]. [cited 2024 Sep 4].

Available from: https://www.ncbi.nlm.nih.gov/pmc/articles/PMC8494593/

28. Anchala R, Kannuri NK, Pant H, Khan H, Franco OH, Di Angelantonio E, et al. Hypertension in India: a systematic review and meta-analysis of prevalence, awareness, and control of hypertension. J Hypertens. 2014 Jun;32(6):1170–7.
29. FR375.pdf [Internet]. [cited 2024 Sep 4]. Available from: https://dhsprogram.com/pubs/pdf/FR375/FR375.pdf
30. Residual lifetime risk for developing hypertension in middle-aged women and men: The Framingham Heart Study - PubMed [Internet]. [cited 2024 Sep 3]. Available from:

https://pubmed.ncbi.nlm.nih.gov/11866648/

31. A Randomized Trial of Intensive versus Standard Blood-Pressure Control | New England Journal of Medicine [Internet]. [cited 2024 Sep 3]. Available from:

https://www.nejm.org/doi/full/10.1056/NEJMoa1511939

32. Global Disparities of Hypertension Prevalence and Control: A Systematic Analysis of Population-based Studies from 90 Countries - PMC [Internet]. [cited 2024 Sep 4]. Available from:

https://www.ncbi.nlm.nih.gov/pmc/articles/PMC4979614/

33. National Health Policy 2017 (English) .pdf [Internet]. [cited 2024 Sep 4]. Available from:

https://nhsrcindia.org/sites/default/files/2021-

07/National%20Health%20Policy%202017%20%28English%29%20.pdf

34. Global action plan for the prevention and control of noncommunicable diseases 2013-2020 [Internet]. [cited 2024 Sep 4]. Available from:

https://www.who.int/publications/i/item/9789241506236

35. Franklin SS, Gustin W, Wong ND, Larson MG, Weber MA, Kannel WB, et al. Hemodynamic patterns of age-related changes in blood pressure. The Framingham Heart Study. Circulation. 1997 Jul 1;96(1):308–15.
36. Yano Y, Reis JP, Colangelo LA, Shimbo D, Viera AJ, Allen NB, et al. Association of Blood

Pressure Classification in Young Adults Using the 2017 American College of Cardiology/American Heart Association Blood Pressure Guideline With Cardiovascular Events Later in Life. JAMA. 2018 Nov 6;320(17):1774–82.

37. Vasan RS, Beiser A, Seshadri S, Larson MG, Kannel WB, D’Agostino RB, et al. Residual lifetime risk for developing hypertension in middle-aged women and men: The Framingham Heart Study. JAMA. 2002 Feb 27;287(8):1003–10.
38. Yusuf S, Hawken S, Ounpuu S, Dans T, Avezum A, Lanas F, et al. Effect of potentially modifiable risk factors associated with myocardial infarction in 52 countries (the INTERHEART study): case-control study. Lancet Lond Engl. 2004 Sep 11;364(9438):937–52.
39. Popkin BM, Adair LS, Ng SW. Global nutrition transition and the pandemic of obesity in developing countries. Nutr Rev. 2012 Jan;70(1):3–21.
40. Cushman WC. Alcohol Consumption and Hypertension. J Clin Hypertens. 2007 May 31;3(3):166–70.
41. Diederichs C, Neuhauser H. The frequency and determinants of blood pressure measurement by a health professional in Germany: A cross-sectional study. Medicine (Baltimore). 2019 Apr;98(16):e15093.
42. Obesity Phenotypes, Diabetes, and Cardiovascular Diseases - PubMed [Internet]. [cited 2024 Sep 3]. Available from: https://pubmed.ncbi.nlm.nih.gov/32437302/
43. Bechthold A, Boeing H, Schwedhelm C, Hoffmann G, Knüppel S, Iqbal K, et al. Food groups and risk of coronary heart disease, stroke and heart failure: A systematic review and dose-response meta-analysis of prospective studies. Crit Rev Food Sci Nutr. 2017 Oct 17;59:00–00.
44. A clinical trial of the effects of dietary patterns on blood pressure. DASH Collaborative Research Group - PubMed [Internet]. [cited 2024 Sep 3]. Available from:

https://pubmed.ncbi.nlm.nih.gov/9099655/

45. Global Burden of Cardiovascular Diseases and Risk Factors, 1990-2019: Update From the GBD 2019 Study - PubMed [Internet]. [cited 2024 Sep 3]. Available from: https://pubmed.ncbi.nlm.nih.gov/33309175/
46. Menke A, Casagrande S, Geiss L, Cowie CC. Prevalence of and Trends in Diabetes Among Adults in the United States, 1988-2012. JAMA. 2015 Sep 8;314(10):1021–9.
47. Lin JD, Chen YL, Wu CZ, Hsieh CH, Pei D, Liang YJ, et al. Identification of Normal Blood Pressure in Different Age Group. Medicine (Baltimore). 2016 Apr 8;95(14):e3188.
48. Salt intake, stroke, and cardiovascular disease: meta-analysis of prospective studies | The BMJ [Internet]. [cited 2024 Sep 3]. Available from: https://www.bmj.com/content/339/bmj.b4567
49. Powles J, Fahimi S, Micha R, Khatibzadeh S, Shi P, Ezzati M, et al. Global, regional and national sodium intakes in 1990 and 2010: A systematic analysis of 24 h urinary sodium excretion and dietary surveys worldwide. BMJ Open. 2013 Dec 20;3:e003733.
50. Mente A, O’Donnell M, Rangarajan S, Dagenais G, Lear S, McQueen M, et al. Associations of urinary sodium excretion with cardiovascular events in individuals with and without hypertension: a pooled analysis of data from four studies. Lancet Lond Engl. 2016 Jul 30;388(10043):465–75.

Response to Reviewers

**Manuscript Title:**

*Hypertension Screening across Different Age Groups in Indian Adults: Evidence from Nationally Representative Cross-Sectional Data*

# Major Revisions Required

## **1. Introduction**

- The introduction should clearly **highlight the research gap** in India’s current hypertension screening policies and how this study aims to fill that gap.
- The authors should briefly discuss existing **global guidelines** on age-based hypertension screening and whether similar studies have been conducted in other **LMICs** (low- and middle-income countries).
- The rationale behind selecting **35 years** as the potential threshold should be better justified in light of previous research.

**Reviewer Comment 1:**

Author Response:

We thank the reviewer for this insightful suggestion. In response, we have substantially revised the Introduction section to better highlight the policy and evidence gap in India’s current hypertension screening practices. Although the National Programme for Prevention and Control of Cancer, Diabetes, Cardiovascular Diseases and Stroke (NPCDCS) recommends annual hypertension screening for adults aged 30 years and above [1], this age threshold has not been empirically validated using stratified national data. Our study directly addresses this gap by analyzing data from NFHS-5, a nationally representative dataset, to determine an evidence-based optimal age for screening using Receiver Operating Characteristic (ROC) analysis.

To place our study in the global context, we have incorporated comparisons with international guidelines. The US Preventive Services Task Force (USPSTF) recommends annual screening for adults aged 40 years and above or for those at higher risk, and screening every 3 to 5 years for low-risk individuals aged 18–39 years [2]. The World Health Organization (WHO) supports routine and opportunistic screening in primary care but does not define a specific age to begin [3]. The International Society of Hypertension (ISH) emphasizes personalized risk-based approaches rather than fixed age thresholds [4].

We also reviewed literature from other LMICs. A nationally representative study from

Nepal found significantly lower hypertension screening rates among individuals aged 15–

29 years, indicating under-screening in younger adults and suggesting a missed opportunity for early diagnosis [5]. However, to our knowledge, no LMIC study has defined a data-driven, age-specific screening cutoff using ROC methods.

To justify the proposed threshold, we now clearly state in the revised manuscript that our analysis showed a marked and statistically significant increase in both systolic and diastolic blood pressure beginning at age 35. Furthermore, ROC analysis identified 36.5 years as the age with the most favorable balance of sensitivity and specificity based on the Youden index. These findings support the selection of 35 years as a practical and evidence-based starting point for routine hypertension screening in the Indian adult population. This recommendation can guide revisions to national policies and align hypertension control efforts with age-specific risk profiles.

References:

1. Ministry of Health and Family Welfare. Module for Multi-Purpose Workers – Prevention, Screening and Control of Common NCDs. 2020. https://main.mohfw.gov.in
2. US Preventive Services Task Force. Screening for Hypertension in Adults: US Preventive Services Task Force Reaffirmation Recommendation Statement. JAMA. 2021;325(16):1650–1656.
3. World Health Organization. HEARTS Technical Package for Cardiovascular Risk Management in Primary Health Care: Risk-based CVD Management. Geneva: WHO; 2020.
4. Unger T, Borghi C, Charchar FJ, et al. 2020 International Society of Hypertension Global Hypertension Practice Guidelines. Hypertension. 2020;75(6):1334–1357.
5. Dhungana RR, Pedisic Z, Dhimal M, et al. Hypertension screening, awareness, treatment, and control: a study of their prevalence and associated factors in a nationally representative sample from Nepal. Global Health Action. 2022;15(1):2000092.

## **2. Methods**

- **Ethical Considerations**: Although NFHS-5 data is publicly available, it is advisable to mention whether any **ethical approval** was sought for secondary data analysis or why it was not required.
- **Confounding Variables**: Were potential confounders (e.g., dietary habits, stress levels, family history of hypertension) excluded due to data limitations? If so, this should be acknowledged.
- **ROC Analysis**: The manuscript lacks detailed reporting on the **ROC curve analysis**.

The authors should provide:

- AUC (Area Under the Curve) values and their **confidence intervals**.
- A **graphical representation** (ROC curve figure) to support the claim that **35 years** is the optimal threshold.
- **Data Handling & Missing Values**: Clarify whether missing data was handled via **imputation or exclusion** and whether it affected the results.

**Author Response:**

We thank the reviewer for these constructive comments. In response, we have revised and elaborated upon the Methods and Results sections as follows:

a) **Ethical Considerations:**

Although NFHS-5 is a publicly available and de-identified dataset, we have included a formal clarification in the *Ethical Approval* section to address this point. Specifically:

“As this study involved secondary analysis of publicly available, anonymized data from the National Family Health Survey (NFHS-5), individual informed consent was not required. However, to ensure institutional compliance and uphold ethical standards, approval was obtained from the Institutional Ethics Committee of PGIMER, Chandigarh (Approval No. IEC-08/2022-2535, dated 17.08.2022).”

b) **Confounding Variables:**

We acknowledge the absence of certain potentially important covariates in the NFHS-5 dataset. These have been explicitly acknowledged in the revised manuscript:

“Potential confounders such as dietary sodium intake, psychological stress levels, and family history of hypertension—well-known determinants of elevated blood pressure—were not collected in NFHS-5 and were therefore not included in the analysis. This limitation has been noted in the Discussion section.”

c) **ROC Analysis – Expanded Reporting:**

We appreciate the recommendation to enhance the reporting of our ROC analysis. The manuscript now includes:

- **AUC Values and Confidence Intervals**:

“The ROC analysis yielded an AUC of **0.71 (95% CI: 0.70–0.72)** for males and **0.68 (95% CI: 0.67–0.69)** for females, indicating a fair ability of age to discriminate individuals with elevated systolic blood pressure (SBP).”

- **Youden Index and Threshold Justification**:

“The Youden Index identified **36.5 years** as the optimal age threshold, offering the best trade-off between sensitivity (57.9%) and specificity (49.7%) in men, with similar performance in women. These values support the recommendation of initiating hypertension screening at 35 years.”

- **ROC Curve Figures**:

We have added **Figures 5 and 6**, which depict the ROC curves for males and females, respectively, and illustrate the diagnostic performance of age as a predictor of elevated BP.

d) **Data Handling and Missing Values:**

We have clarified our approach to missing data as follows:

“Data were analyzed using complete case analysis (listwise deletion). Participants with missing values in outcome or independent variables were excluded from relevant statistical models. No imputation was performed. The sample size varied slightly across analyses and has been reported in all relevant tables to ensure transparency.”

## **3. Results**

- **Statistical Reporting:**
  - The results should explicitly mention **Odds Ratios (ORs) with Confidence Intervals (CIs)** for each predictor.
  - A **table summarizing the AUC values, sensitivity, and specificity** of different age thresholds should be included.
- **Figures and Tables:**
  - Consider adding a **visualization** of hypertension prevalence by age group.
  - If possible, include a **decision curve analysis** to illustrate the impact of different screening thresholds.

**Author Response:**

We thank the reviewer for this valuable feedback. In response, we have made the following revisions and additions to strengthen the Results section:

Odds Ratios with 95% Confidence Intervals:

We now explicitly report adjusted Odds Ratios (ORs) along with their 95% Confidence Intervals (CIs) for all significant predictors of elevated systolic and diastolic blood pressure, as shown in the updated Tables 3 and 4. These findings are also summarized in the narrative text under the Results section to ensure clarity and interpretability.

Summary of ROC Metrics – Table 7:

We have added Table 7, which presents the key diagnostic performance metrics of age as a predictor of hypertension. This includes:

Table 7 presents the diagnostic performance of age as a predictor of hypertension using ROC analysis for both systolic and diastolic blood pressure in males and females. The analysis identifies 36.5 years as the optimal cut-off age for initiating hypertension screening in both sexes, based on the Youden Index. Among males, the area under the curve (AUC) was 0.71 for systolic BP and 0.69 for diastolic BP, indicating fair discriminatory ability. In females, the AUC values were slightly lower at 0.68 for systolic BP and 0.66 for diastolic BP. Sensitivity and specificity values at this cut-off were reasonably balanced, ranging from 56% to 61% sensitivity and 47% to 50% specificity. These findings support 35–36 years as a data-driven and practical threshold for routine hypertension screening among Indian adults.

Table 7: ROC-Based Diagnostic Performance of Age for Predicting Hypertension in Indian

Adults (NFHS-5, 2019–21)

| **Sex** | **Outcome** | **Cut-off**  **Age**  **(years)** | **AUC**  **(95% CI)** | **Sensitivity (%)** | **Specificity (%)** | **Youden Index** |
| --- | --- | --- | --- | --- | --- | --- |
| **Male** | SBP ≥140 mmHg | 36.5 | 0.71 (0.70–  0.72) | 57.9 | 49.7 | 0.076 |
|  | DBP ≥90 mmHg | 36.5 | 0.69 (0.68–  0.70) | 56.5 | 50.2 | 0.067 |
| **Female** | SBP ≥140 mmHg | 36.5 | 0.68 (0.67–  0.69) | 60.6 | 47.4 | 0.08 |
|  | DBP ≥90 mmHg | 36.5 | 0.66 (0.65–  0.67) | 57.8 | 47.6 | 0.054 |

Visual Trends in Blood Pressure by Age – Figures 1 to 4:

We have incorporated Figures 1–4 to visualize the age-wise trends in systolic and diastolic blood pressure separately for male and female participants. These figures clearly show a sharp increase in both SBP and DBP beginning from the age group of 35–39 years, reinforcing our empirical basis for selecting this threshold.

Decision Curve Analysis:

While we agree that decision curve analysis (DCA) can offer valuable insight into the clinical utility of screening thresholds, we were unable to perform DCA in the current analysis due to the cross-sectional nature and scope limitations of the NFHS-5 dataset, which does not capture longitudinal outcomes or decision-related consequences. However, we have acknowledged this limitation in the Discussion section and recommended DCA as a direction for future prospective cohort studies.

**4. Discussion**  **Comparison with Existing Research:**

- Strengthen the discussion by comparing findings with hypertension screening recommendations from organizations such as the **World Health**

**Organization (WHO)** or **American Heart Association (AHA)**.

- How do these findings align with or contradict previous Indian studies on hypertension screening?
- **Public Health and Policy Implications:**
  - The manuscript should provide **clear policy recommendations**. Should India’s national screening guidelines be revised to start at 35 years?
  - Are there specific **subpopulations** (e.g., rural vs. urban, male vs. female) that would benefit more from early screening?
- **Limitations:**
  - Acknowledge that **self-reported behaviors (e.g., alcohol, smoking, physical activity)** may be underreported in NFHS-5.
  - Discuss the **cross-sectional nature** of the study, limiting causal inferences. o	Mention potential **regional disparities** in hypertension screening practices within India.

**Author Response:**

We are grateful to the reviewer for this comprehensive and insightful feedback. In response, we have substantially revised the *Discussion* section to incorporate the following:

**Comparison with Existing Guidelines and Research:**

We have now elaborated on both global and national hypertension screening guidelines:

- The **World Health Organization (WHO)** promotes opportunistic and routine blood pressure screening in adults but does not prescribe a specific starting age, instead emphasizing integration into primary care systems [1].
- The **US Preventive Services Task Force (USPSTF)** recommends annual hypertension screening for adults aged ≥40 years, or earlier for individuals with risk factors. For low-risk adults aged 18–39, screening is advised every 3–5 years [2].
- In India, the **NPCDCS guidelines** advise annual screening starting at age 30 [3], but this threshold lacks direct support from empirical, age-stratified analyses using national-level data.

Our study fills this critical gap by identifying **36.5 years** as the optimal age for initiating screening, based on ROC analysis of NFHS-5 data. This is the first Indian study to derive such a threshold from a nationally representative sample. Prior research, including the study by **Prenissl et al. (2019)**, has highlighted poor awareness and control rates of hypertension in India but has not addressed the age at which screening should ideally begin [4]. Our findings thus provide a valuable addition to both Indian and global literature on evidence-based screening age thresholds.

**Public Health and Policy Implications:**

To translate these findings into actionable recommendations, we have revised the *Discussion* and *Conclusion* to include the following:

“Based on the ROC-derived thresholds and observed age-related trends in blood pressure, we recommend that India’s national guidelines revise the starting age for routine hypertension screening to **35 years** for both males and females. This age captures the point at which blood pressure begins to rise substantially, and balances early detection with screening efficiency.” Furthermore, we highlight the need for **subpopulation-specific strategies**:

- **Urban males** and adults with **elevated waist-hip ratios** exhibited significantly higher odds of hypertension and may benefit from **earlier or more frequent screening intervals**.
- **Women with diabetes or obesity** are another high-risk group for whom earlier intervention may be warranted.
- In **rural and underserved regions**, enhanced community outreach and mobile screening programs could help mitigate barriers to access and awareness.

**Limitations:**

The *Limitations* section has been expanded to include the following:

- “Self-reported variables in NFHS-5, including alcohol consumption, smoking, and physical activity, may be prone to **social desirability bias** and underreporting, potentially attenuating the observed associations.”
- “Due to the **cross-sectional** nature of NFHS-5, the study cannot establish causal relationships between risk factors and elevated blood pressure. **Longitudinal studies** are necessary to validate our proposed screening threshold.”
- “We acknowledge potential **regional disparities** in hypertension awareness, screening uptake, and access to care across Indian states and districts, which may affect the generalizability of our recommendations. Future subnational analyses are recommended.” **References:**

1. World Health Organization. *HEARTS Technical Package: Risk-Based Cardiovascular Disease (CVD) Management*. Geneva: WHO; 2020. <https://www.who.int/publications/i/item/9789240001367>
2. US Preventive Services Task Force. Screening for Hypertension in Adults: US Preventive Services Task Force Reaffirmation Recommendation Statement. *JAMA*. 2021;325(16):1650–1656.
3. Ministry of Health and Family Welfare, India. *Module for Multi-Purpose Workers – Prevention, Screening and Control of Common NCDs*. 2020.
4. Prenissl J, Manne-Goehler J, Jaacks LM, et al. Hypertension screening, awareness, treatment, and control in India: A nationally representative cross-sectional study among individuals aged 15–49 years. *PLoS Med*. 2019;16(5):e1002801.

## **5. Conclusion**

- The conclusion should **emphasize actionable recommendations** based on the study findings.
- The need for **longitudinal studies** to confirm the 35-year threshold should be mentioned.
- If available, suggest **intervention strategies** to improve screening uptake among younger adults.

**Author Response:**

We thank the reviewer for this important recommendation. In response, we have revised the *Conclusion* section to incorporate the following elements:

- **Actionable Policy Recommendation**:

We now emphasize the key takeaway that, based on nationally representative ROC analysis, **hypertension screening should ideally begin at age 35** for both men and women in India. This represents a meaningful, evidence-based update to current guidelines that can enhance early detection and improve long-term cardiovascular outcomes.

- **Need for Longitudinal Validation**:

We have acknowledged that the cross-sectional nature of the NFHS-5 dataset limits causal inference and that **longitudinal cohort studies** are needed to further validate 35 years as the optimal screening threshold.

- **Intervention Strategies**:

We have added practical suggestions for improving screening uptake among younger adults, particularly:

- **Community-based awareness campaigns** targeting the 30–45 age group. o	**Integration of BP screening into workplace wellness programs**.
- **Mobile-based digital health reminders** and use of **ASHAs and frontline health workers** to expand screening in underserved areas.

These additions aim to ensure the conclusion is policy-relevant, forward-looking, and aligned with public health implementation goals.

# Minor Revisions & Language Edits

- Ensure that all abbreviations (e.g., NFHS-5, AUC) are **defined upon first use**.
- Improve **flow and coherence** in the introduction and discussion sections.
- The manuscript would benefit from a **professional language edit** to enhance readability and conciseness.

Thank you for the comments have modified it.
